# ACTL6A depletion induces KLF4-mediated anti-tumorigenic effects in colorectal cancer

**DOI:** 10.1038/s41419-025-07946-w

**Published:** 2025-08-28

**Authors:** Hye-Ju Yang, Eun-Ju Kim, Yeonsoo Kim, Youngwon Cho, Younghee Choi, Sang-Hyun Song, Tae-You Kim

**Affiliations:** 1 Department of Molecular Medicine and Biopharmaceutical Sciences, Graduate School of Convergence Science and Technology, Seoul National University, Seoul, Republic of Korea; 2 Cancer Genomics Research Laboratory, Cancer Research Institute, Seoul National University, Seoul, Republic of Korea; 3 Department of Internal Medicine, Seoul National University Hospital, Seoul, Republic of Korea

**Keywords:** Chromatin remodelling, Apoptosis

## Abstract

ACTL6A, a subunit of the SWI/SNF and INO80 chromatin remodeling complexes, is frequently overexpressed in various cancers, and its depletion attenuates cell proliferation in colorectal cancer (CRC). However, the epigenetic mechanisms underlying ACTL6A function remain poorly understood. Here, we aimed to elucidate how ACTL6A regulates chromatin accessibility and gene expression in CRC. Integrated multi-omics analyses revealed that ACTL6A deficiency alters chromatin accessibility and upregulates P53 pathway-related genes, accompanied by the recruitment of SWI/SNF and INO80 complexes. Mechanistically, ACTL6A depletion enhances KLF4 binding at newly accessible regions, where it cooperates with these chromatin remodeling complexes to activate P53 pathway-related genes and induce apoptosis. ACTL6A contributes to CRC cell proliferation by inhibiting the KLF4-mediated transcriptional activation of tumor-suppressive genes. Thus, our findings highlight that targeting ACTL6A may serve as a promising therapeutic strategy in CRC.

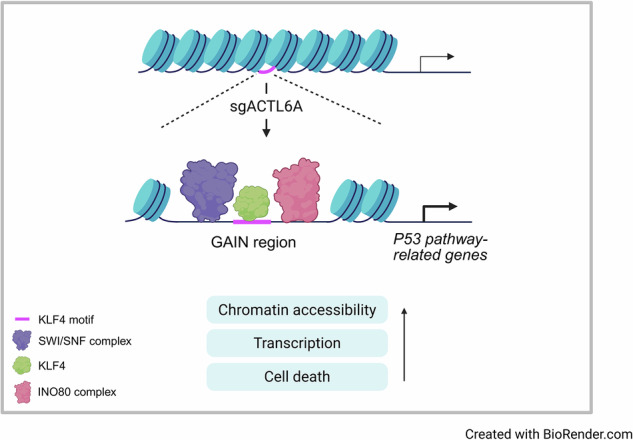

## Introduction

Epigenetic mechanisms, including histone modification, DNA methylation, and nucleosome remodeling, play a pivotal role in cell development and differentiation by regulating gene expression [1, 2]. The dysregulation of these processes is a hallmark of cancer [2–4]. In particular, chromatin accessibility is fundamental to transcription, DNA replication, and repair [5]. ATP-dependent chromatin remodeling complexes use ATP hydrolysis to modulate histone–DNA interactions within nucleosomes, generating nucleosome-depleted regions at promoters and enhancers [6]. This modification facilitates transcription factor (TF) binding, promoting gene expression by enabling transcriptional complexes to access regulatory regions [6–9]. Additionally, chromatin remodeling complexes regulate chromatin accessibility to allow RNA polymerase II (RNA PII) to achieve precise control over gene expression patterns [8].

ATP-dependent chromatin remodeling complexes are categorized into four families SWI/SNF (BAF), INO80, ISWI, and CHD [1, 9, 10]. Mutations in these complexes are prevalent in human cancers, markedly contributing to tumorigenesis and cancer progression [9, 11]. For example, mutations in the SWI/SNF complexes occur in >20% of all human cancers [12–17], and INO80-complex mutations have also been identified in various cancers [12, 18–20]. The deletion or mutation of subunits within these complexes can result in either apoptosis or tumorigenesis, depending on factors like cell type and the specific subunit involved [1], highlighting the need to explore the therapeutic implications of subunit depletion.

ACTL6A (also known as BAF53A and INO80K) is a shared subunit of the SWI/SNF and INO80 complexes and functions as a driver of colorectal cancer (CRC), glioma, squamous cell carcinoma (SCC), hepatocellular carcinoma [21–24]. ACTL6A promotes metastasis and cell proliferation in hepatocellular carcinoma and head and neck SCC and plays a pivotal role in tumor progression and patient survival outcomes by activating the Notch and Hippo–YAP pathways [22, 24]. Elevated ACTL6A levels activate SCC-related genes by recruiting SWI/SNF and TEAD–-YAP complexes to chromatin [25]. Conversely, ACTL6A loss reduces cell proliferation and tumor growth [25, 26]. Thus, as ACTL6A is a shared subunit of the SWI/SNF and INO80 complexes, its depletion may disrupt chromatin dynamics and gene expression. However, the precise epigenetic mechanisms by which its repression affects CRC cell proliferation remain unclear.

In this study, we aimed to elucidate the epigenetic mechanisms by which ACTL6A contributes to CRC cell proliferation. We investigated how ACTL6A depletion alters chromatin accessibility and modulates the genomic recruitment of SWI/SNF and INO80 chromatin remodeling complexes. The role of KLF4 as a key mediator of these chromatin changes was further investigated, with a particular focus on its contribution to the activation of P53 pathway-related genes. Additionally, the regulatory mechanism of KLF4 expression and the potential clinical relevance of ACTL6A and KLF4 expression in CRC were examined. We hypothesized that ACTL6A promotes tumor progression in CRC by suppressing KLF4-mediated transcriptional activation of tumor-suppressive gene programs, particularly those linked to the P53 pathway.

## Results

### ACTL6A expression is upregulated in CRC and regulates CRC cell proliferation

**Fig. 1:**
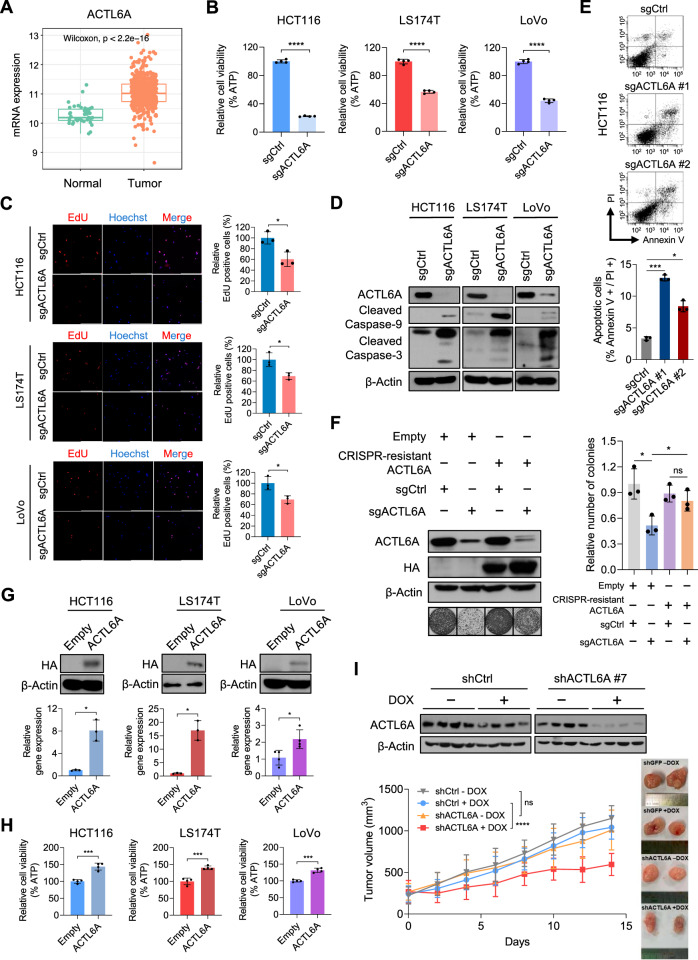
ACTL6A expression is upregulated in CRC and regulates CRC cell proliferation. **A** mRNA expression data for colon adenocarcinoma (COAD) were obtained from the Cancer Genome Atlas (TCGA) database to compare the differences in *ACTL6A* mRNA expression between normal and tumor samples. Expression values were determined using variance stabilizing transformation and the DESeq2 package. **B** Relative cell viability of HCT116, LS174T, and LoVo cells transduced with sgCtrl or sgACTL6A. Cell viability was determined using an ATP assay. ATP levels (y-axis) were measured using CellTiter-Glo. *n* = 4 biological replicates. ****p < 0.0001. **C** EdU assay of HCT116, LS174T, and LoVo cells transduced with sgCtrl (sgControl) or sgACTL6A to detect cell proliferation. Representative fluorescent images of EdU and Hoechst (left) staining and the proportion of EdU-positive cells (right). *n* = 3 biological replicates. Scale bar: 200 μm. *p < 0.05. **D** Western blotting with the indicated antibodies in HCT116, LS174T, and LoVo cells transduced with sgCtrl or sgACTL6A. β-Actin was used as the loading control. **E** Annexin V/propidium iodide assay for apoptosis in HCT116 cells transduced with sgCtrl or two independent sgRNAs targeting ACTL6A. Representative flow cytometry plots (top) and statistical analysis (bottom). *n* = 3 biological replicates. *p < 0.05, ***p < 0.001. **F** Western blotting with the indicated antibodies in HCT116 cells expressing empty vector or clustered regularly interspaced short palindromic repeats (CRISPR)-resistant ACTL6A on day 6 after transduction with sgCtrl or sgACTL6A. β-Actin was used as the loading control (top left). Colony formation assay for cells with representative images (bottom left) and statistical analysis (right) are shown. *n* = 3 biological replicates. *p < 0.05, ***p < 0.001, ns, not significant. **G** Western blotting with the indicated antibodies in HCT116, LS174T, and LoVo cells transduced with an empty or HA-tagged ACTL6A-expressing vector. β-Actin was used as the loading control (top). Normalized *ACTL6A* mRNA expression in HCT116, LS174T, and LoVo cells transduced using an empty or an HA-tagged *ACTL6A*-expressing vector was measured using quantitative reverse transcription-polymerase chain reaction (RT-qPCR) (bottom). *n* = 3 biological replicates for HCT116 and LS174T; *n* = 4 biological replicates for LoVo. *p < 0.05. **H** Relative cell viability of HCT116, LS174T, and LoVo cells transduced using an empty or HA-tagged ACTL6A-expressing vector. Cell viability was determined using an ATP assay. ATP levels (y-axis) were measured using CellTiter-Glo. *n* = 4 biological replicates. ***p < 0.001. **I** Western blotting of the indicated antibodies in HCT116 cells transduced using shCtrl or shACTL6A after doxycycline (DOX) treatment. β-Actin was used as the loading control (top left). Tumor growth curves for the four groups of mice with or without DOX treatment (bottom left), and representative tumor images (right). HCT116 cells stably transduced with DOX-inducible shCtrl or shACTL6A were implanted in the flanks of nude mice. The mice were randomly allocated to four groups 1 week after tumor implantation (n = 13–15 mice per group). The DOX-treated group was administered DOX (1 mg/mL) and 5% sucrose in drinking water. Tumor volume was measured using calipers at the indicated time points. Data are presented as the mean tumor volume ± standard error of the mean. A two-way analysis of variance with Tukey’s test was performed for multiple comparisons. ****p < 0.0001, ns, not significant.

The Cancer Genome Atlas (TCGA) colon adenocarcinoma (COAD) data were analyzed to explore the transcription of *ACTL6A* in CRC. *ACTL6A* expression was higher in CRC tissues than in normal colon tissues (Fig. 1A). The distribution of *ACTL6A* copy numbers among patients was then examined to investigate the relationship between *ACTL6A* overexpression and copy number variation in CRC. Samples were categorized according to copy numbers as LOSS (<2) or GAIN (>2) and diploid. Notably, 42.9% of CRC cases showed copy number amplification (≥3 copies), while 56.3% exhibited no alterations (Supplementary Fig. 1A, B). Higher *ACTL6A* expression was noted in GAIN samples than in LOSS samples (Supplementary Fig. 1C). However, the difference in *ACTL6A* expression between the diploid and GAIN groups was not significant, suggesting that overexpression may occur independent of copy number gain (Supplementary Fig. 1D). Moreover, organoids derived from patients with CRC (PDOs) demonstrated higher *ACTL6A* levels than the matched normal colon organoids (Supplementary Fig. 2A), confirming that *ACTL6A* was markedly overexpressed in CRC tissues.

To investigate the functional role of ACTL6A in CRC cells, we generated *ACTL6A*-knockout cells using the clustered regularly interspaced short palindromic repeats (CRISPR)-Cas9 system. ACTL6A depletion significantly reduced cell viability, as indicated by decreased intracellular ATP levels (Fig. 1B). EdU incorporation assays revealed a marked decrease in proliferating cells, suggesting that ACTL6A is essential for cell proliferation (Fig. 1C). Furthermore, apoptotic markers such as cleaved caspase-3 and -9 were upregulated following ACTL6A depletion, indicating apoptotic signaling pathway activation (Fig. 1D). Consistent with these findings, annexin V/propidium iodide staining followed by flow cytometry revealed a substantial increase in the apoptotic cell population upon ACTL6A depletion (Fig. 1E). ACTL6A knockdown using two distinct short hairpin RNAs (shRNAs) also suppressed cell proliferation (Supplementary Fig. 2B). In addition, ACTL6A depletion in the CRC PDOs led to a substantial reduction in tumor cell viability (Supplementary Fig. 2C, D). Notably, ACTL6A depletion inhibited cell proliferation in CRC cell lines but had minimal effects on CCD-18Co normal colon fibroblasts (Supplementary Fig. 2E).

CRISPR-resistant ACTL6A vectors harboring two nucleotide mutations with unaltered amino acid sequences were generated to definitively attribute cell death to ACTL6A depletion (Supplementary Fig. 2F). Ectopic CRISPR-resistant ACTL6A expression rescued the proliferation defect caused by CRISPR-Cas9-mediated ACTL6A depletion, confirming that proliferation arrest was functionally dependent on ACTL6A (Fig. 1F). In contrast, ACTL6A overexpression promoted cell growth in CRC cell lines and normal colon organoids (Fig. 1G, H, Supplementary Fig. 2G). These findings indicate that ACTL6A is crucial for cancer cell growth and survival.

A mouse xenograft model with HCT116 cells harboring ACTL6A-targeting shRNA was established to explore the role of ACTL6A in tumor growth in vivo. Doxycycline (DOX)-induced ACTL6A knockdown substantially reduced tumor volume (Fig. 1I), indicating the inhibitory effect of ACTL6A depletion on tumor growth. These findings were further validated in vitro using a colony formation assay (Supplementary Fig. 2H); ACTL6A was highly expressed in CRC and played a pivotal role in promoting cell proliferation in vitro and in vivo.

### ACTL6A depletion alters gene expression and chromatin accessibility linked to the P53 pathway

**Fig. 2:**
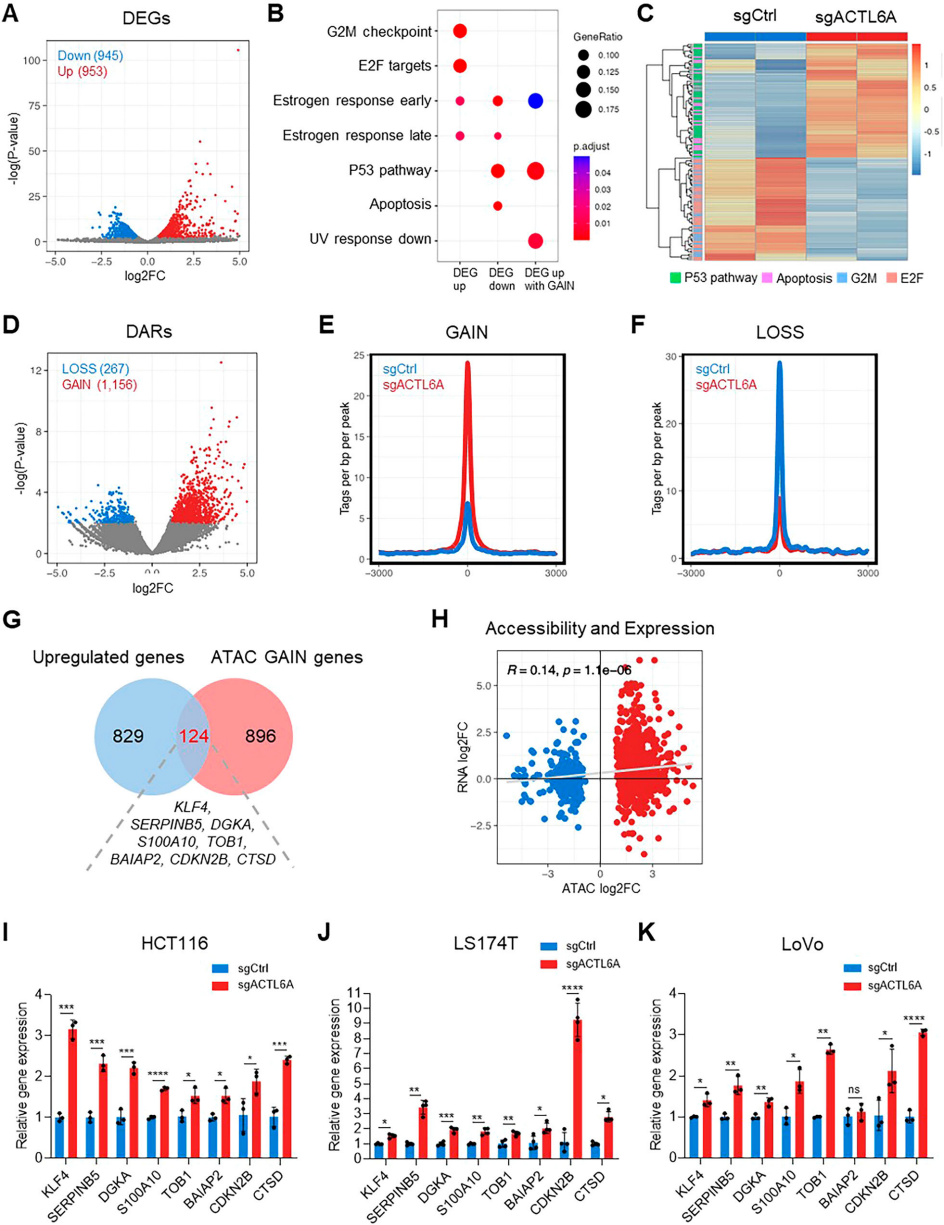
ACTL6A depletion alters gene expression and changes chromatin accessibility linked to the P53 pathway. **A** Differentially expressed genes (DEGs) were identified based on |FC | > 1.5 and false discovery rate (FDR) < 0.05 after ACTL6A depletion in HCT116 cells compared with the wild type (WT). **B** Gene set functional enrichment analysis using gProfiler2. The inputs included the upregulated DEGs, genes with GAIN regins in their closest regions, and their overlaps. **C** Heatmap of RNA-seq data (n = 2) from HCT116 cells after ACTL6A depletion compared with the WT based on the log2(TPM + 1) values. Each row indicates genes associated with the E2F targets, G2M checkpoint, apoptosis, and P53 pathways. **D** Differentially accessible regions (DARs) were identified based on |FC | > 2 and *p* < 0.01 after ACTL6A depletion in HCT116 cells compared with the WT using assay for transposase-accessible chromatin sequencing (ATAC-seq) (n = 3). **E** Histogram demonstrating tags per bp per peak from −3000 bp upstream to +3000 bp downstream of the identified GAIN after ACTL6A depletion in HCT116 cells compared with the WT, based on ATAC-seq (n = 3). **F** Histogram demonstrating tags per bp per peak from −3000 bp upstream to +3000 bp downstream of the identified LOSS after ACTL6A depletion in HCT116 cells compared with the WT, based on ATAC-seq (n = 3). **G** Venn diagram illustrating the overlap between the upregulated genes (RNA Up) and the genes closest to ATAC GAIN regions. **H** Correlation plot illustrating changes in accessibility and expression. Red and blue indicate GAIN and LOSS, respectively. Pearson’s method was used to calculate the correlation coefficients. **I**–**K** Normalized mRNA expression of the indicated genes in HCT116, LS174T, and LoVo cells transduced with sgCtrl or sgACTL6A was measured using RT-qPCR. n = 3 biological replicates for HCT116 and LoVo cells, n = 4 biological replicates for LS174T cells. *p < 0.05, **p < 0.01, ***p < 0.001, ****p < 0.0001; ns, not significant.

RNA sequencing (RNA-seq) analysis was performed using biological duplicates (n = 2) to elucidate transcriptomic landscape changes underlying the inhibition of proliferation in ACTL6A-depleted HCT116 cells. Differential expression analysis revealed 945 downregulated and 953 upregulated genes (false discovery rate < 0.05, fold change > 1.5) (Fig. 2A). Functional enrichment analysis using the Hallmark gene set from the Molecular Signatures Database (MsigDB) indicated that the upregulated genes were enriched in pathways such as the “P53 pathway” and “apoptosis.” In contrast, downregulated genes were enriched in “G2M checkpoint” and “E2F targets” (Fig. 2B, C). These findings potentially reflect the decreased cell proliferation after in vitro and in vivo ACTL6A depletion (Fig. 1).

The effects of ACTL6A depletion on P53-deficient (*TP53*^*−/−*^) HCT116 cells were further investigated because of the pivotal role of the P53 pathway in regulating cell death and proliferation. ACTL6A depletion in *TP53*^*−/−*^ HCT116 cells reduced cell proliferation (Supplementary Fig. 3A, B). Unexpectedly, RNA-seq analysis revealed increased P53 pathway-related gene expression upon ACTL6A depletion despite the absence of P53 (Supplementary Fig. 3C), suggesting that ACTL6A may regulate these genes through P53-independent mechanisms. Additionally, apoptosis-related genes were markedly upregulated in the ACTL6A-depleted *TP53*^*−/−*^ cells (Supplementary Fig. 3D). Notably, the differentially expressed genes (DEGs) identified between ACTL6A-depleted *TP53* wild-type (WT) cells and controls showed a correlation with those in *TP53*^*−/−*^ HCT116 cells (Supplementary Fig. 3E). These results indicate that ACTL6A depletion modulates gene expression independent of P53, further supporting the role of ACTL6A in tumor suppression mechanisms that are not solely dependent on P53.

Assay for transposase-accessible chromatin sequencing (ATAC-seq) was performed using biological triplicates (n = 3) to examine the effect of ACTL6A deficiency on chromatin accessibility. Our analysis revealed 1,423 differentially accessible regions (DARs) in ACTL6A-depleted and control HCT116 cells, with 1,156 regions showing enhanced chromatin accessibility (GAIN) and 267 regions demonstrating decreased accessibility (LOSS) (FDR < 0.01, fold change > 2) (Fig. 2D–F). Similar overall patterns between control and ACTL6A-depleted conditions were observed when examining the genome-wide read density across all replicates in merged peak regions (Supplementary Fig. 4A).

An independent Gene Expression Omnibus dataset (GSE156786) including ATAC-seq data from FaDu cells with small interfering RNA-induced ACTL6A knockdown was used to validate the identified DARs to determine whether the chromatin accessibility changes in the ACTL6A-depleted HCT116 cells were consistent across different cell types and ACTL6A depletion methods. Specifically, ATAC-seq read density from the GSE156786 dataset was analyzed at the DARs defined in HCT116 cells. The analysis revealed that chromatin accessibility at the GAIN regions—previously defined upon ACTL6A depletion in HCT116 cells—increased progressively with the extent of ACTL6A reduction. By contrast, chromatin accessibility at LOSS regions remained largely unchanged following ACTL6A reduction in FaDu cells (Supplementary Fig. 4B). These results highlight the consistency of GAIN regions across different cell types and ACTL6A depletion conditions, suggesting that ACTL6A exerts a conserved regulatory effect on these regions, whereby its loss consistently promotes chromatin accessibility across multiple cellular contexts.

Integrated RNA- and ATAC-seq data analysis was performed to elucidate the relationship between the changes in chromatin landscape and ACTL6A depletion-induced cell death. The genes corresponding to the nearest transcription start sites for DARs in the ATAC-seq data were annotated using Homer [27]. Notably, 124 genes related to the GAIN regions were markedly upregulated in the RNA-seq analysis (Fig. 2G), and functional enrichment analysis showed that these genes were enriched in the “P53,” “estrogen response early,” and “UV response down” pathways (Fig. 2B). Alterations in chromatin accessibility were significantly correlated with RNA expression changes (*R* = 0.14, *p* < 1.1 × 10^–6^; Fig. 2H). The enhanced expression of *KLF4*, *SERPINB5*, *DGKA*, *S100A10*, *TOB1*, *BAIAP2*, *CDKN2B*, and *CTSD* was validated by quantitative reverse transcription-polymerase chain reaction (RT-qPCR) using three CRC cell lines: HCT116, LS174T, and LoVo (Fig. 2I–K).

### ACTL6A depletion alters SWI/SNF and INO80 binding, leading to chromatin accessibility changes

**Fig. 3:**
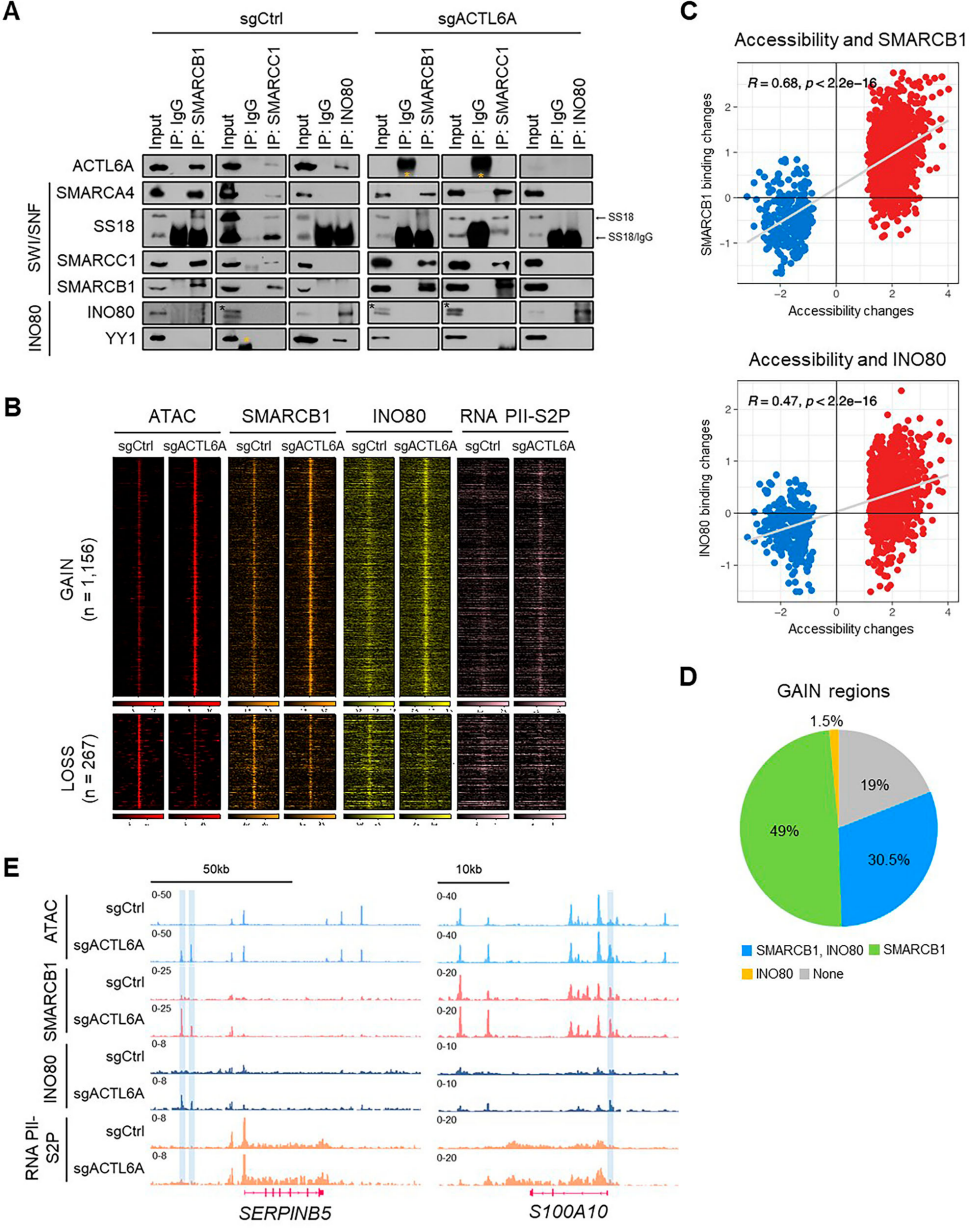
ACTL6A loss alters SWI/SNF and INO80 binding, leading to chromatin accessibility changes. **A** Co-immunoprecipitation (co-IP) experiments using antibodies against SMARCB1, SMARCC1, and INO80 in whole-cell extracts from HCT116 ells transduced with sgCtrl or sgACTL6A. Western blotting results of ACTL6A (SWI/SNF and INO80 complex subunit), SMARCA4, SS18, SMARCC1, SMARCB1 (SWI/SNF complex subunits), INO80, and YY1 (INO80 complex subunits). Arrows indicate SS18 and SS18/IgG. IgG bands are marked by orange asterisks (*). The black asterisk (*) indicates the INO80 band. **B** Heatmaps of GAIN and LOSS regions following ACTL6A depletion, showing ATAC-seq, SMARCB1, INO80, KLF4, and RNA Pol II-S2P ChIP-seq signals. **C** Correlation plot indicating the change in accessibility and SMARCB1 (top) and INO80 (bottom) enrichment. Red and blue indicate the GAIN and LOSS regions, respectively. Pearson’s method was used to calculate correlation coefficients. **D** Pie chart illustrating the distribution of SMARCB1 and INO80 enrichment in the GAIN regions. **E** IGV tracks of SMARCB1, INO80, RNA PII-S2P enrichment (ChIP-seq) and chromatin accessibility (ATAC-seq) at the *SERPINB5* and *S100A10* loci in HCT116 cells transduced with sgCtrl or sgACTL6A.

The composition of SWI/SNF and INO80 complexes after ACTL6A depletion was examined to determine whether their assembly was altered. Co-immunoprecipitation (co-IP) of SMARCB1 and SMARCC1—the core subunits of the SWI/SNF complex [11]—pulled down SMARCA4, SS18, and ACTL6A in control cells (Fig. 3A). In ACTL6A-depleted cells, co-IP of SMARCB1 and SMARCC1 continued to pull down SMARCA4 and SS18, indicating that the assembly and stability of the SWI/SNF complex were maintained despite ACTL6A loss. In parallel, co-IP of the INO80 complex in control cells recovered known subunits, including YY1 and ACTL6A; however, the interaction between INO80 and YY1 was diminished upon ACTL6A depletion, suggesting a potential role of ACTL6A in stabilizing this association. Notably, no physical interaction was detected between the SWI/SNF and INO80 complexes, as co-IP of SMARCB1, SMARCC1, and INO80 did not reveal inter-complex association.

We investigated whether ACTL6A depletion altered genomic localization of the SWI/SNF and INO80 complexes and whether differential chromatin accessibility was due to the differential binding of these complexes. Chromatin immunoprecipitation (ChIP)-seq for SMARCB1 and INO80—the subunits of their respective complexes—was conducted using biological duplicates (n = 2) to analyze their binding profiles after ACTL6A depletion. SMARCB1 and INO80 binding was enhanced in the GAIN regions where chromatin accessibility was increased, whereas decreased binding was observed in the LOSS regions with reduced accessibility (Fig. 3B). Additionally, correlation analysis revealed significant positive correlations between the alterations in accessibility and changes in SMARCB1 and INO80 binding at the DARs (*R* = 0.64, *R* = 0.47, *p* < 2.2 × 10^–16^; Fig. 3C), indicating that the changes in chromatin accessibility were closely associated with the chromatin-remodeling activity of the SWI/SNF and INO80 complexes.

To further explore the relationship between INO80 and YY1 following ACTL6A depletion, we examined their co-binding dynamics at the GAIN regions using ChIP-seq profiles generated from ACTL6A-depleted cells (INO80, n = 2; YY1, n = 1). Despite the reduced physical interaction between INO80 and YY1 (Fig. 3A), the proportion of GAIN regions co-occupied by both factors increased from 3.5% to 10.7% upon ACTL6A loss (Supplementary Fig. 4C), suggesting local redistribution rather than a global loss of co-binding. While the total number of genome-wide co-bound peaks slightly decreased from 6,446 to 5,659, the number of YY1- and INO80-only peaks at GAIN regions increased from 5.4% to 9.3% and 4.5% to 21.0%, respectively, in ACTL6A-depleted cells (Supplementary Fig. 4C, D). These findings suggest that ACTL6A depletion modulates the genomic binding pattern of INO80 and YY1, potentially by altering chromatin accessibility or recruiting co-factors, and that co-binding at regulatory elements can occur in a context-dependent manner independent of direct physical interaction.

RNA PII regulates transcription by interacting with chromatin remodeling complexes [28–30]. Ser2 phosphorylation of the RNA PII C-terminal domain (RNA PII-S2P) indicates sustained transcriptional activity and promotes the recruitment of other Pol II elongation-related factors [31–33]. Thus, a ChIP-seq analysis of RNA PII-S2P was performed using biological duplicates (n = 2); RNA PII-S2P binding increased in the GAIN regions and decreased in the LOSS regions (Fig. 3B, Supplementary Fig. 4E). A significant positive correlation was noted between changes in chromatin accessibility and RNA PII binding, suggesting that ACTL6A depletion-induced alterations in chromatin accessibility affected RNA PII-mediated gene expression (*R* = 0.33, *R* = 0.47, *p* < 2.2 × 10^–16^; Supplementary Fig. 4F).

Genomic distribution analysis revealed that >92% of the GAIN and LOSS regions were in distal regulatory elements outside the promoters (Supplementary Fig. 4G), suggesting that changes in chromatin accessibility primarily affected distal regulatory regions such as enhancers. Additionally, an analysis of SMARCB1 and INO80 binding sites within the GAIN regions revealed that 30.5% of these sites were co-occupied by both factors, while 49% and 1.5% were bound by SMARCB1 or INO80 alone, respectively (Fig. 3D). This result suggests that the SWI/SNF complex can cooperate with INO80 or function independently to enhance chromatin accessibility in certain GAIN regions, albeit without any physical interaction (Fig. 3A). For instance, a considerable increase in chromatin accessibility at the distal regulatory elements of P53 pathway-related genes (e.g., *SERPINB5* and *S100A10*) was observed, accompanied by increased SMARCB1 and INO80 binding at these sites. Furthermore, RNA PII-S2P enrichment was increased across gene bodies after ACTL6A depletion (Fig. 3E). These findings suggest that ACTL6A deficiency modulates chromatin accessibility by altering SWI/SNF and INO80 complex binding, potentially activating the P53 pathway-related genes through RNA PII.

### ACTL6A depletion induces KLF4 expression and recruitment to accessible chromatin regions

**Fig. 4:**
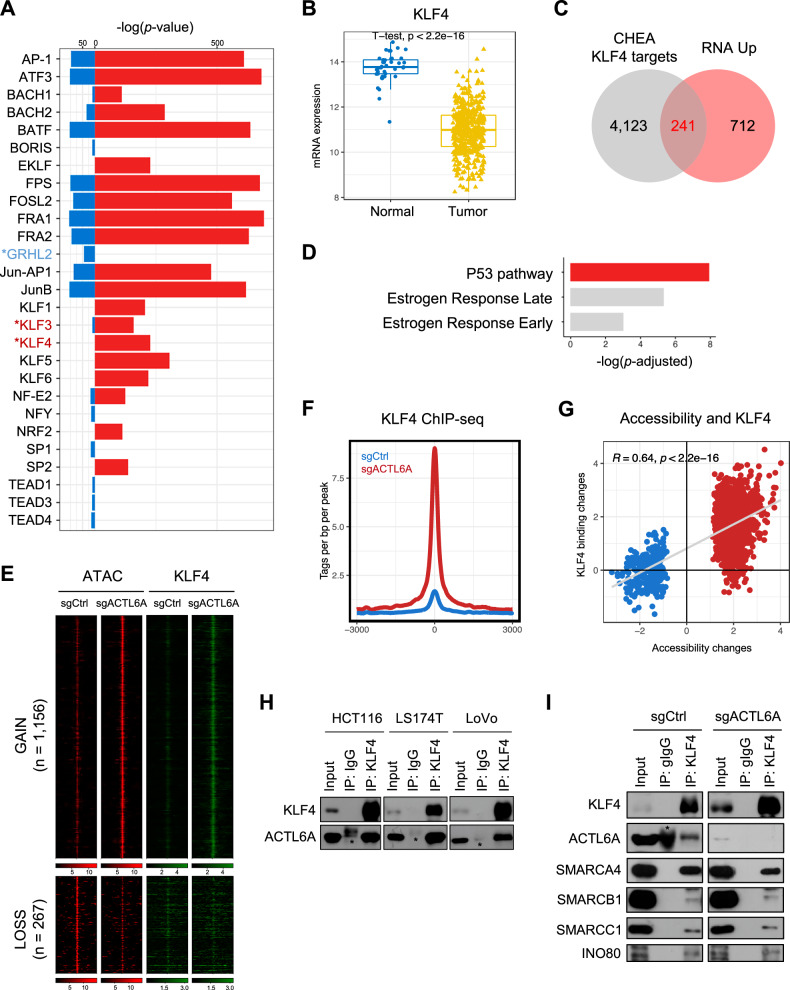
ACTL6A depletion induces KLF4 expression and recruitment to accessible chromatin regions. **A** Bar plot illustrating the results of motif analysis. Red and blue indicate the GAIN and LOSS, respectively. Asterisks (*) indicate the DEGs. **B** TCGA-COAD datasets were used to compare the differences in *KLF4* expression between matched normal and tumor tissues. Expression values were determined using VST with the DESeq2 package. **C** Venn diagram illustrating the overlap between the KLF4 target genes based on the ChIP Enrichment Analysis database and the upregulated genes induced by ACTL6A depletion. **D** The overlapping genes shown in (**C**) were used for functional enrichment analysis based on the Hallmark gene set from the Molecular Signatures Database. **E** Heatmaps of GAIN and LOSS regions following ACTL6A depletion, showing KLF4 ChIP-seq signals. **F** Histogram illustrating KLF4 tags per base pair per peak from −3000 bp upstream to +3000 bp downstream of the identified GAIN regions upon ACTL6A depletion in HCT116 cells. **G** Correlation plot illustrating changes in accessibility and KLF4 enrichment. Red and blue indicate the GAIN and LOSS, respectively. Pearson’s analysis was used to calculate the correlation coefficients. **H** Co-IP experiments using the KLF4 antibody on whole-cell extracts from HCT116, LS174T, and LoVo cells. Western blotting results of KLF4 and ACTL6A. IgG bands are marked by black asterisks (*). **I** Co-IP experiments using KLF4 antibody in whole-cell extracts from HCT116 cells transduced with sgCtrl or sgACTL6A. Western blotting results of KLF4, ACTL6A (SWI/SNF and INO80 complex subunit), SMARCA4, SMARCB1, SMARCC1 (SWI/SNF complex subunits), INO80 (INO80 complex subunit). IgG bands are marked by black asterisks (*).

Motif analysis was performed using Homer [27] to determine potential TFs that recognize and bind to the GAIN regions; the top 20 motifs for the GAIN and LOSS regions were identified (Fig. 4A, Supplementary Fig. 5A, B). AP-1 family motifs were identified in both regions, while KLF and TEAD family motifs were predominantly observed in the GAIN and LOSS regions, respectively. These results were validated by analyzing genome-wide chromatin accessibility at regions containing KLF3, KLF4, GRHL2, and TEAD motifs. As expected, regions containing KLF3 and KLF4 motifs showed increased accessibility, whereas those containing GRHL2 and TEAD motifs showed decreased accessibility (Supplementary Fig. 5C, D). We next analyzed the expression of TFs corresponding to the identified motifs. *KLF3* and *KLF4* expression were upregulated, whereas that of *GRHL2* was downregulated. No significant changes were noted in *TEAD1/3/4* expression (Supplementary Fig. 5E).

We focused on KLF4 as a key TF associated with GAIN regions. *KLF4* mRNA expression in the TCGA-COAD dataset was examined to investigate the role of KLF4 in colon tissue samples; its expression was lower in tumor tissues than in normal tissues (Fig. 4B), supporting its potential relevance in CRC. To assess the transcriptional activity of KLF4 upon ACTL6A depletion, we compared upregulated genes with known KLF4 targets using the ChIP Enrichment Analysis database [34]. A substantial proportion of the upregulated genes (25.29%, 241/953) overlapped with KLF4 target genes (Fig. 4C). Functional enrichment analysis of these 241 overlapping genes revealed the significant enrichment of P53 pathway-related genes (Fig. 4D).

To validate these predictions and determine whether KLF4 directly binds to GAIN regions, we performed KLF4 ChIP-seq under ACTL6A-deficient conditions. This analysis confirmed that KLF4 binding was indeed enhanced at GAIN regions in ACTL6A-depleted cells (Fig. 4E, F). Furthermore, correlation analysis demonstrated a positive relationship between changes in chromatin accessibility and KLF4 binding to DARs (*R* = 0.64, *p* < 2.2 × 10^–16^; Fig. 4G). These findings suggest that ACTL6A depletion promotes chromatin opening at KLF4 motif-containing regions, facilitating increased KLF4 binding and the transcriptional activation of target genes, including those involved in the P53 pathway.

To investigate the molecular relationship between ACTL6A and KLF4, we performed co-IP assays under basal conditions in three CRC cell lines, demonstrating physical interaction between the two proteins (Fig. 4H). Given the potential cooperation between KLF4 and chromatin remodeling complexes at GAIN regions, we further examined whether KLF4 physically associates with these complexes. Indeed, KLF4 was found to interact with subunits of both the SWI/SNF (SMARCA4, SMARCC1, and SMARCB1) and INO80 (INO80) chromatin remodeling complexes (Supplementary Fig. 5F). To assess whether these interactions depend on ACTL6A, we performed co-IP assays following ACTL6A depletion. Notably, KLF4 maintained its association with both SWI/SNF and INO80 components regardless of ACTL6A status (Fig. 4I), indicating that ACTL6A is not required for the physical interaction between KLF4 and chromatin remodeling complexes. However, ACTL6A loss markedly enhanced the binding of KLF4, SWI/SNF, and INO80 to GAIN regions (Figs. 3B, 4F). These observations suggest that rather than mediating complex formation, ACTL6A regulates the genomic targeting of the KLF4–chromatin remodeler complex. Taken together, these findings imply that in the absence of ACTL6A, KLF4 is more efficiently recruited to accessible chromatin regions in cooperation with remodeling complexes.

### ACTL6A suppresses KLF4-mediated activation of P53 pathway genes to promote cell survival

**Fig. 5:**
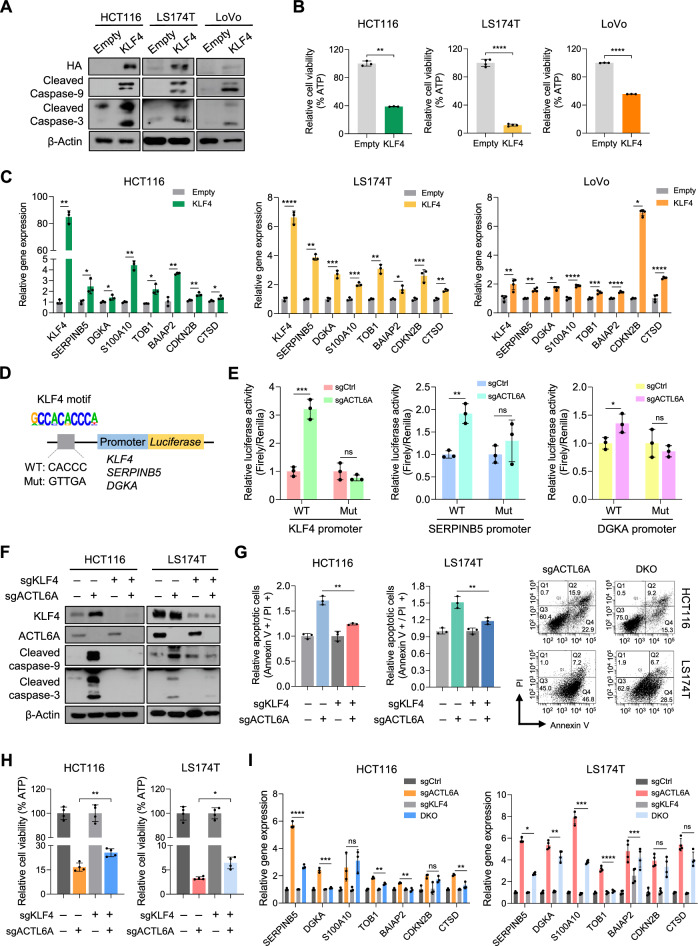
ACTL6A suppresses KLF4-mediated activation of P53 pathway genes to promote cell survival. **A** Western blotting of the indicated antibodies in HCT116, LS174T, and LoVo cells transduced with an empty or HA-tagged KLF4-expressing vector. β-Actin was used as the loading control. **B** Relative cell viability of HCT116, LS174T, and LoVo cells transduced with an empty or HA-tagged KLF4-expressing vector. Cell viability was determined using an ATP assay. ATP levels (y-axis) were measured using CellTiter-Glo. n = 3 biological replicates for HCT116 and LoVo, n = 4 biological replicates for LS174T. **p < 0.01, ****p < 0.0001. **C** Normalized mRNA expression of P53 pathway-related genes in HCT116, LS174T, and LoVo cells transduced with an empty or HA-tagged KLF4-expressing vector. n = 3 biological replicates for HCT116 and LS174T, n = 4 biological replicates for LoVo. *p < 0.05, **p < 0.01, ***p < 0.001, ****p < 0.0001. **D** Schematic of luciferase reporter constructs containing wild-type (WT) or mutant (Mut) KLF4 binding motifs upstream of *KLF4*, *SERPINB5*, or *DGKA* promoters cloned into pGL3-basic vector. **E** Dual luciferase reporter assays were performed to assess *KLF4*, *SERPINB5*, or *DGKA* promoter activity in HCT116 cells transduced with sgCtrl or sgACTL6A. Firefly luciferase activity was normalized to *Renilla* luciferase to control for transfection efficiency. n = 3 biological replicates. *p < 0.05, **p < 0.01, ***p < 0.001. **F** Western blotting using the indicated antibodies in HCT116 and LS174T cells transduced with indicated sgRNAs. β-Actin was used as the loading control. **G** Annexin V/propidium iodide assay for apoptosis in HCT116 and LS174T cells transduced with indicated sgRNAs. Statistical analysis (left) and representative flow cytometry plots (right) of sgACTL6A or sgKLF4+sgACTL6A (DKO) (right). n = 3 biological replicates. ** *p* < 0.01. **H** Relative cell viability of HCT116 and LS174T transduced with indicated sgRNAs. Cell viability was determined using an ATP assay. ATP levels (y-axis) were measured using CellTiter-Glo. n = 4 biological replicates. *p < 0.05, ** *p* < 0.01. **I** Normalized mRNA expression of the indicated genes in HCT116 and LS174T cells transduced with indicated sgRNAs, as determined by RT-qPCR. n = 3 biological replicates for HCT116, n = 4 biological replicates for LS174T. *p < 0.05, **p < 0.01, ***p < 0.001, ns, not significant.

As KLF4 mediates tumor-suppressive effects following ACTL6A depletion, we examined whether ectopic KLF4 expression could reproduce these phenotypes in CRC cells [35, 36]. Ectopic KLF4 expression enhanced cleaved caspase-3 and -9 levels (Fig. 5A), indicating enhanced apoptosis and marked cell proliferation inhibition (Fig. 5B). In addition, KLF4 overexpression elevated the expression of P53 pathway-related genes, including *SERPINB5*, *DGKA*, *S100A10*, *TOB1*, *BAIAP2*, *CDKN2B*, and *CTSD*, which were also upregulated upon ACTL6A depletion (Fig. 5C). These findings suggest that KLF4 acts as a key regulator of the tumor-suppressive transcription induced by ACTL6A loss.

To further support the regulatory link between ACTL6A and KLF4, we analyzed gene expression datasets from TCGA and the Genotype-Tissue Expression (GTEx) project. Interestingly, *ACTL6A* expression was negatively correlated with KLF4 expression in CRC tissues (*R* = − 0.36, *p* = 5.6 × 10^–19^; Supplementary Fig. 6A). Moreover, KLF4 showed a positive correlation with P53 pathway-related genes (*R* = 0.28, *p* = 1.3 × 10^–11^; Supplementary Fig. 6B).

To determine whether ACTL6A regulates the transcriptional activity of KLF4 and its downstream targets, we performed luciferase reporter assays using constructs containing either WT or mutant (Mut) KLF4 binding motifs upstream of the *KLF4*, *SERPINB5*, and *DGKA* promoters (Fig. 5D). ACTL6A loss significantly enhanced the promoter activity of these genes in the presence of WT but not Mut motifs, indicating that this effect depends on KLF4-mediated transcriptional regulation (Fig. 5E). These findings demonstrate that ACTL6A loss facilitates KLF4-dependent transcriptional activation, particularly for genes involved in the P53 pathway.

To validate the hypothesis that enhanced KLF4 activity induced by ACTL6A loss contributes to cell death and the activation of P53 pathway-related genes, double knockout (DKO) models were generated by deleting KLF4 followed by ACTL6A in HCT116 and LS174T cells. ACTL6A depletion in KLF4-deficient cells resulted in reduced apoptosis and attenuated the decrease in cell viability compared to that seen after ACTL6A depletion in KLF4-proficient cells (Fig. 5F–H). Moreover, the upregulation of several P53 pathway-related genes upon ACTL6A depletion was significantly attenuated when ACTL6A was depleted in KLF4-deficient cells in both HCT116 and LS174T cells (Fig. 5I), indicating that KLF4 is essential for mediating the transcriptional and apoptotic responses triggered by ACTL6A loss. Collectively, these results highlight that ACTL6A promotes cell survival by repressing the activity of KLF4.

### KLF4 mediates chromatin remodeling in cooperation with SWI/SNF and INO80 upon ACTL6A loss

**Fig. 6:**
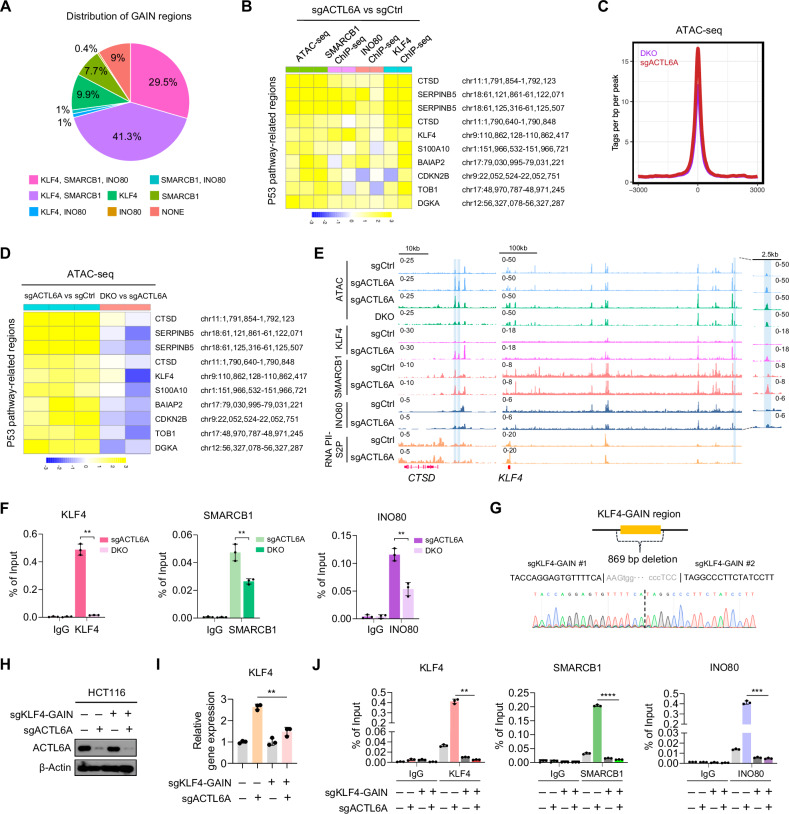
KLF4 mediates chromatin remodeling in cooperation with SWI/SNF and INO80 upon ACTL6A loss. **A** Pie chart illustrating the distribution of SMARCB1, INO80, and KLF4 binding sites based on ChIP-seq data. **B** Heatmaps revealing alterations in normalized tag values for P53 pathway-related GAIN regions upon ACTL6A depletion in HCT116 cells. The first three columns represent the ATAC-seq replicates (n = 3, green), followed by two columns for the SMARCB1 ChIP-seq replicates (n = 2, light purple), two colums for INO80 ChIP-seq replicates (n = 2, light coral), and two columns for KLF4 ChIP-seq replicates (n = 2, aqua). Changes in the normalized tag values were calculated as log2(sgACTL6A + 1) − log2(sgCtrl + 1). **C** Histogram revealing the ATAC-seq tags per base pair per peak from −3000 bp upstream to +3000 bp downstream of the identified GAIN regions in the HCT116 cells transduced with sgACTL6A or sgKLF4+sgACTL6A (DKO). **D** Heatmaps showing the changes in normalized tag values for the P53 pathway-related GAIN regions upon ACTL6A depletion in HCT116 cells. The first three columns represent the ATAC-seq replicates for log2(sgACTL6A + 1) − log2(sgCtrl + 1), whereas the next two columns show the ATAC-seq replicates for log2(DKO + 1) − log2(sgACTL6A + 1). **E** IGV tracks of SMARCB1, INO80, KLF4, and RNA PII-S2P enrichment (ChIP-seq) and chromatin accessibility (ATAC-seq) at the *CTSD* and *KLF4* loci in HCT116 cells transduced with indicated sgRNAs. **F** ChIP-qPCR for KLF4, SMARCB1 and INO80 at the GAIN region upstream of the *KLF4* (KLF4-GAIN) in HCT116 cells transduced with sgACTL6A or sgKLF4+sgACTL6A (DKO). n = 3 biological replicates. **p < 0.01. **G** Sanger sequencing showing a 869 bp deletion in KLF4-GAIN region, two sgRNA target sequences (black and gray text), and a protospacer adjacent motif (PAM) sequence (lowercase). **H** Western blotting results of ACTL6A in HCT116 cells transduced with indicated sgRNAs. β-Actin was used as the loading control. **I** Normalized mRNA expression of *KLF4* in HCT116 cells transduced with indicated sgRNAs. n = 3 biological replicates. *p < 0.05, **p < 0.01, ****p < 0.0001. **J** ChIP-qPCR for KLF4, SMARCB1 and INO80 at the GAIN region upstream of the *KLF4* (KLF4-GAIN) in HCT116 cells transduced with indicated sgRNAs. n = 3 biological replicates. **p < 0.01, ***p < 0.001, ****p < 0.0001.

To investigate whether KLF4 is involved in regulating chromatin accessibility following ACTL6A depletion, we analyzed the distribution of SWI/SNF, INO80, and KLF4 binding sites within GAIN regions. A substantial overlap was observed between GAIN regions and the binding sites of SMARCB1, INO80, and KLF4 (Fig. 6A). Notably, heatmaps of GAIN regions located near P53 pathway-related genes showed increased binding of both SMARCB1 and INO80 (Fig. 6B), indicating that many of these regions are co-regulated by SMARCB1, INO80, and KLF4.

To assess the functional contribution of KLF4 to chromatin remodeling upon ACTL6A depletion, we performed ATAC-seq (n = 2). If KLF4 were not involved, ACTL6A loss would be expected to increase chromatin accessibility regardless of KLF4 status. However, chromatin accessibility within the GAIN regions did not increase upon ACTL6A depletion in the absence of KLF4, suggesting that KLF4 is required for the chromatin remodeling induced by ACTL6A loss (Fig. 6C). Similarly, in GAIN regions proximal to P53 pathway-related genes, the increase in accessibility observed with ACTL6A depletion alone was markedly reduced in DKO cells (Fig. 6D). Gene tracks for *CTSD* and *KLF4* further illustrated that the increase in chromatin accessibility observed upon ACTL6A depletion was attenuated in DKO cells (Fig. 6E), indicating that KLF4 is essential for chromatin remodeling at GAIN regions. Notably, KLF4 also bound to the GAIN region upstream of its own gene, suggesting a positive feedback mechanism through which KLF4 may reinforce its own transcription and maintain chromatin remodeling at GAIN regions following ACTL6A loss.

To determine whether KLF4 contributes to the recruitment of chromatin remodeling complexes at GAIN regions upon ACTL6A depletion, we performed ChIP-qPCR for KLF4, SMARCB1, and INO80 at the GAIN region upstream of the *KLF4* gene (hereafter referred to as the KLF4-GAIN region). The binding of all three factors was significantly reduced in DKO cells, suggesting that KLF4 is required for the recruitment of SWI/SNF and INO80 complexes (Fig. 6F), in line with the reduced chromatin accessibility observed in DKO cells, further supporting the role of KLF4 in facilitating chromatin remodeling at GAIN regions.

Given that ACTL6A depletion increased chromatin accessibility at the KLF4-GAIN region and promoted strong co-binding of KLF4, SMARCB1, and INO80, along with the upregulation of KLF4 expression, we sought to determine whether this region functions as a regulatory element essential for KLF4 transcriptional activation. To assess its functional significance, we deleted the KLF4-GAIN region using CRISPR-Cas9 and subsequently depleted ACTL6A (Fig. 6G, H). Although ACTL6A loss alone upregulated KLF4 expression, this induction was substantially attenuated in cells lacking the KLF4-GAIN region (Fig. 6I). Moreover, ChIP-qPCR revealed that ACTL6A loss in KLF4-GAIN region-depleted cells impaired the binding of KLF4, SMARCB1, and INO80, supporting the role of this region as a functional element that mediates KLF4 activation upon ACTL6A depletion (Fig. 6J).

Collectively, these findings suggest that ACTL6A suppresses *KLF4* expression by preventing the recruitment of SWI/SNF and INO80 complexes to its upstream regulatory region. Upon ACTL6A depletion, KLF4 is transcriptionally induced and cooperates with these chromatin remodeling complexes at GAIN regions to promote chromatin accessibility, activate P53 pathway-related genes, and induce cell death.

Finally, analysis of the TCGA-COAD dataset revealed a trend suggesting that patients with high ACTL6A and low KLF4 expression exhibit poorer survival outcomes compared to those with low ACTL6A and high KLF4 expression (*p* = 0.1) (Supplementary Fig. 6C). Although this finding does not meet the conventional threshold for statistical significance, it suggests a potential inverse relationship between ACTL6A and KLF4 expression and CRC prognosis.

## Discussion

This study identifies ACTL6A as a pivotal epigenetic regulator of chromatin accessibility and transcriptional repression in CRC through its inhibitory effect on KLF4, a tumor-suppressive TF. We demonstrate that ACTL6A contributes to CRC cell proliferation by modulating the transcriptional and chromatin remodeling activity of KLF4 at P53 pathway-related genes. Through integrated multi-omics approaches, we show that ACTL6A depletion increases chromatin accessibility at distal regions associated with P53 pathway-related gene regulation and facilitates the recruitment of KLF4 in cooperation with SWI/SNF and INO80 complexes, leading to apoptotic cell death. These findings highlight ACTL6A as a promising therapeutic target for CRC.

ACTL6A overexpression is associated with CRC proliferation, invasion, and metastasis [23, 26]. Consistently, TCGA-COAD samples showed higher ACTL6A mRNA expression than normal colon tissues (Fig. 1A). Interestingly, this overexpression was not always accompanied by copy number gain, suggesting that mechanisms beyond gene amplification, such as epigenetic, transcriptional, or post-transcriptional regulation, may drive ACTL6A upregulation [37–40].

Notably, our findings revealed that P53 pathway-related genes were upregulated independently of P53 (Supplementary Fig. 3). P53 is a key tumor suppressor that regulates cell death and the cell cycle [41]. ACTL6A depletion in *TP53*^*−/−*^ cells induced cell death and P53 pathway gene upregulation comparable to that in *TP53* WT cells, supporting P53-independent apoptosis [42, 43]. KLF4, a tumor suppressor in CRC [35, 36, 44, 45], is also known to drive P53-independent cell death [46]. In addition, P53 inhibition in ACTL6A-depleted cells did not block p21Cip1 induction, further supporting a P53-independent mechanism [47]. Collectively, these results suggest that ACTL6A depletion activates P53 pathway-related genes independently of P53, potentially offering therapeutic opportunities in P53-deficient cancers. Interestingly, although this activation occurs regardless of P53 status, the degree of transcriptional induction was more pronounced in *TP53*^*−/−*^ cells (Supplementary Fig. 3C). This difference may be explained by the regulatory role of P53 in wild-type cells, where P53-mediated survival checkpoints and feedback mechanisms may limit excessive transcriptional activation. In contrast, the absence of these regulatory constraints in *TP53*^*−/−*^ cells may allow a stronger KLF4-mediated transcriptional response. These findings suggest that while the pathway is fundamentally P53-independent, the transcriptional amplitude may vary depending on P53 status, potentially reflecting differences in cellular regulatory environments.

Although ACTL6A depletion does not substantially alter the core composition of the SWI/SNF complex, it disrupts interactions within the INO80 complex, particularly affecting the INO80 subunit YY1 (Fig. 3A). This suggests that ACTL6A is crucial for stabilizing interactions within the INO80 complex, particularly between INO80 and YY1. Co-binding analysis revealed that despite reduced physical interaction between INO80 and YY1 upon ACTL6A loss, the proportion of GAIN regions co-occupied by both factors increased (Supplementary Fig. C, D). This suggests that ACTL6A depletion promotes the context-dependent redistribution of chromatin remodelers, possibly through increased chromatin accessibility or recruitment by co-factors such as KLF4. However, as the chromatin binding of INO80 and YY1 is influenced by multiple regulatory inputs beyond KLF4, the observed co-binding changes likely represent one of several potential mechanisms, and further studies will be required to clarify the precise molecular basis.

Our data support KLF4 as a regulator of chromatin remodeling and transcriptional activation following ACTL6A loss. Motif enrichment, ATAC-seq, and ChIP-seq analyses revealed that KLF4 binds to ACTL6A-dependent GAIN regions, where it colocalizes with SWI/SNF and INO80 complexes. Mechanistically, we found that ACTL6A physically interacts with KLF4. To our knowledge, no study has reported a direct interaction between ACTL6A and KLF4. To examine whether ACTL6A modulates the interaction between KLF4 and chromatin remodeling complexes, we performed KLF4 co-IP under ACTL6A-depleted conditions. Interestingly, KLF4 remained associated with both SWI/SNF and INO80 components regardless of ACTL6A status (Fig. 4I), indicating that ACTL6A does not block the physical interaction between KLF4 and chromatin remodelers. Instead, ACTL6A may interfere with their genomic targeting or functional recruitment to regulatory regions. This observation supports a model in which ACTL6A limits the ability of KLF4 to engage in chromatin remodeling.

The functional relevance of the ACTL6A–KLF4 axis was further validated using DKO models (Figs. 5, 6). ACTL6A depletion induced apoptosis and reduced cell viability, but these effects were significantly attenuated by KLF4 deletion, confirming the essential role of KLF4 in mediating the apoptotic response. The partial rescue observed in DKO models suggests that other transcription factors may also contribute to this regulatory network. Supporting this notion, motif analysis following ACTL6A depletion identified AP-1, TEAD, and GRHL2 as potential additional contributors (Supplementary Fig. 5A–E). AP-1 motifs, enriched in both GAIN and LOSS regions, are known to interact with chromatin remodelers to modulate chromatin structure and accessibility [48]. In contrast, TEAD and GRHL2 motifs were predominantly enriched in LOSS regions. This is consistent with previous findings that ACTL6A depletion reduces chromatin accessibility at TEAD-binding sites [25]. GRHL2, frequently upregulated in CRC [49], also showed reduced accessibility upon ACTL6A loss, suggesting that ACTL6A broadly coordinates oncogenic transcriptional networks. Whether these transcription factors directly cooperate with chromatin remodelers or function through parallel pathways remains to be elucidated.

Importantly, KLF4 regulates its own transcription via a distal GAIN region. CRISPR-mediated deletion of this element reduced KLF4, SMARCB1, and INO80 binding and diminished KLF4 upregulation after ACTL6A depletion (Fig. 6G–J), supporting a positive feedback loop. Overall, ACTL6A restricts KLF4 activity by suppressing its transcription and limiting access to upstream regulatory elements.

Despite these insights, this study has limitations. Multi-omics analyses were conducted in HCT116 cells, and further validation in other CRC models is needed. In addition, although ACTL6A depletion led to a clear reduction in tumor growth in vitro, these findings may not fully recapitulate the complexity of in vivo conditions, where additional regulatory mechanisms and interactions could influence the observed effects.

In conclusion, our findings define ACTL6A as an epigenetic regulator that suppresses both KLF4 expression and its ability to activate transcriptional programs. Upon ACTL6A depletion, KLF4 recruits SWI/SNF and INO80 complexes to GAIN regions near P53 pathway-related genes, leading to increased chromatin accessibility and transcriptional activation of tumor-suppressive targets. This activation of cell death-associated programs contributes to the reduction in cell viability observed upon ACTL6A loss. Targeting ACTL6A may therefore reactivate silenced tumor-suppressive gene networks through KLF4, offering a promising epigenetic therapeutic strategy for CRC.

## Materials and methods

### Cell culture and transfection

Human CRC cell lines (HCT116, LS174T, LoVo, and SW480) were obtained from the Korean Cell Line Bank (Seoul, Republic of Korea), and the normal colon fibroblast line CCD-18Co (CRL-1459) from the American Tissue Culture Collection (Manassas, VA, USA). HCT116 *P53*^*−/−*^ cells were generated using the CRISPR-Cas9 system and confirmed by Sanger sequencing (Supplementary Fig. 3A). Cells were cultured in RPMI 1640 (#LM011-01; WELGENE, Gyeongsan-si, Republic of Korea) supplemented with 10% fetal bovine serum (#S001-01; WELGENE) and gentamicin (10 μg/mL) at 37 °C in a 5% CO₂-humidified atmosphere. Cell lines were authenticated by short tandem repeat profiling and regularly tested for mycoplasma contamination.

Lentiviral vectors carrying sgRNAs were produced in 293FT cells by transfecting plasmids with ViraPower lentiviral packaging mix (Thermo Fisher Scientific, Waltham, MA, USA) as previously described [50]. Retroviral vectors were transfected into 293FT cells using pUMVC (#84490; Addgene, Watertown, MA, USA) and pCMV-VSV-G (#8454; Addgene). Viruses were harvested after 48 h and transduced into target cells with 6 μg/mL polybrene (#H9268; Sigma-Aldrich, St. Louis, MO, USA). After 24 h, cells were selected with 1 μg/mL puromycin (#P8833; Sigma-Aldrich) or 5 μg/mL blasticidin (#15205; Sigma-Aldrich) for 6 days. Gene knockout or overexpression was validated by western blotting or RT-qPCR. Single cells were plated into 96-well plates for clonal selection, and silencing or overexpression was confirmed after clonal expansion.

### Plasmids

For gene knockout, sgRNAs targeting each gene or the control (GFP) were cloned into the LentiCRISPRv2 vector (#52961; Addgene). TRC lentiviral shRNAs for ACTL6A (TRCN0000072273, TRCN0000072274; Horizon) were used for ACTL6A knockdown and to generate DOX-inducible clones in Tet-pLKO-puro plasmids (#21915; Addgene). pBABE-puro (#1764; Addgene) and MSCV-PIG (#18751; Addgene) were used to overexpress HA-tagged ACTL6A, subcloned from pBS-hBAF53a (#17879; Addgene). A CRISPR-resistant ACTL6A clone was generated using the QuickChange-II site-directed mutagenesis kit (#200523; Agilent Technologies, Santa Clara, CA, USA). The LentiCRISPRv2-blast vector (#98293; Addgene) was used to deplete ACTL6A in cells expressing CRISPR-resistant ACTL6A. pBpuro-HA KLF4 FL (#34589; Addgene) was used for KLF4 overexpression. All plasmids were validated by Sanger sequencing. CRISPR-Cas9 sgRNAs and shRNA primers are listed in Supplementary Table 1. The pGL3 basic vector (#212936; Addgene) was used for the luciferase reporter assay.

### TCGA-COAD data analysis

TCGA-COAD expression and clinical data were downloaded using the TCGAbiolinks package. Gene expression counts were normalized with the DESeq2 package, and expression values were calculated by variance-stabilizing transformation. For survival analysis, tumor samples with ACTL6A expression higher than the maximum level in normal tissues were classified as the ACTL6A High group, and those equal to or lower than this maximum were classified as the ACTL6A Low group. Tumor samples with KLF4 expression lower than the minimum level in normal tissues were classified as the KLF4 Low group, and those equal to or higher than this minimum were classified as the KLF4 High group. Kaplan–Meier survival curves were generated, and log-rank tests were performed to compare survival rates between the ACTL6A High/KLF4 Low and ACTL6A Low/KLF4 High groups. Survival probabilities and the number of patients at risk were visualized using Kaplan–Meier plots.

### PDO culture

PDOs were established and cultured as previously described [51]. Organoid medium was refreshed three times per week with the following: 50% Wnt-3a conditioned media (for normal organoids only), 10% R-spondin1 conditioned media, 10% Noggin conditioned media or 100 ng/mL recombinant Noggin (#120-10 C; Peprotech, Cranbury, NJ, USA), 50 ng/mL recombinant human EGF (#AF-100-15; Peprotech), 1× B27 (#12587010; Gibco, Waltham, MA, USA), 1.25 mM n-acetyl cysteine (#A9165; Sigma-Aldrich), 3 μM SB202190 (#S6067; Sigma-Aldrich), 500 nM A83-01 (#2939; Tocris, Bristol, UK), 10 nM prostaglandin E2 (P5640; Sigma-Aldrich), 10 nM gastrin (G9145; Sigma-Aldrich), and 100 μg/mL primo (#ant-pm-1; Invitrogen, Waltham, MA, USA) in basal media (advanced DMEM/F12 [#12634010; Gibco]) supplemented with 1× penicillin/streptomycin (#15140122; Gibco), 10 mM HEPES (#15630080; Gibco), and 1× Glutamax (#35050061; Gibco).

### Cell viability assay

A cell viability assay was performed using CellTiter-Glo 3D (#G9682; Promega, Madison, WI, USA), according to the manufacturer’s protocol. Cells (2000–4000 cells/well) were seeded in a 96-well plate.

### Colony formation assay

A colony formation assay was performed 6 d after viral transduction. Cells were plated in triplicate at a density of 1 × 10^4^ cells/well in 6-well plates. The medium was removed after 7 d, and the cells were fixed and stained with Coomassie Brilliant Blue solution (0.1% Coomassie Brilliant Blue R-250, 50% methanol, and 10% acetic acid). Cells were washed five times in phosphate-buffered saline (PBS) and once in distilled water.

### Flow cytometry

Cells (1 × 10^6^) were stained using an FITC Annexin V Apoptosis Detection Kit II (#556570; BD Biosciences, Franklin Lakes, NJ, USA) according to the manufacturer’s instructions. Stained cells were immediately subjected to flow cytometry using a FACSCanto™ II Flow Cytometer (BD Biosciences).

### EdU incorporation assay

Cell proliferation was evaluated using the Click-iT™ Plus EdU Alexa Fluor™ 594 Imaging Kit (#C10646; Invitrogen). All cells were treated with 10 μM EdU and processed according to the manufacturer’s staining protocol. Captured images were analyzed using ImageJ software. The number of EdU-positive cells was determined based on Hoechst nuclear staining and expressed as a percentage of the total number of cells per field.

### Luciferase reporter assay

Plasmid constructs used in the assays were generated by subcloning into the empty pGL3 basic vector. Promoter–luciferase reporter constructs were generated by PCR-amplifying genomic regions upstream of the transcriptional start sites of KLF4, SERPINB5, and DGKA from HCT116 genomic DNA using Q5 high-fidelity DNA polymerase (#M0491L; New England Biolabs, Ipswich, MA, USA). PCR products were digested with BglII and HindIII and inserted upstream of the luciferase gene. Vectors containing WT or Mut KLF4-binding motifs were constructed by cloning the respective motifs upstream of the *KLF4*, *SERPINB5*, or *DGKA* promoter regions between the KpnI and NheI restriction sites. All constructs were verified by sequencing. Luciferase assays were performed 48 h after transfection using a Dual-Luciferase Reporter Assay System (#E1910; Promega, Madison, WI, USA) following the manufacturer’s instructions. Relative luciferase activity was measured and normalized to *Renilla* luciferase activity.

### RNA extraction, RT-qPCR, and RNA-seq

Cells were harvested 6 d after viral transduction. Total RNA was extracted using TRI Reagent (#TR-118; Molecular Research Center, Cincinnati, OH, USA). Total RNA (2 μg) was reverse transcribed for cDNA synthesis, and real-time qPCR analysis was performed as previously described [52]. Primer sequences used for RT-qPCR are listed in Supplementary Table 2. Sequencing libraries were generated according to the standard protocol of Illumina (San Diego, CA, USA) for high-throughput sequencing. The transcriptome was then sequenced using a Genome Analyzer IIx (Illumina) as previously described [53].

### Western blotting and co-IP assay

Cells were washed twice with PBS and collected. Whole-cell lysates were prepared by incubating cells on ice for 30 min in cell lysis buffer (50 mM Tris-HCl [pH 7.5], 150 mM NaCl, 1% NP-40, 0.1% Na-deoxycholate, 50 mM NaF, 1 mM sodium pyrophosphate, 1 mM EDTA, and protease/phosphatase inhibitors), followed by centrifugation at 16,022 × *g* at 4 °C for 20 min. The supernatant was collected, and protein concentrations were measured using a BCA assay (Thermo Fisher Scientific). Equal amounts of protein were separated by 10% SDS-PAGE and transferred to nitrocellulose membranes (Cytiva, Marlborough, MA, USA). Membranes were blocked with 1× TBS-T (20 mM Tris-HCl [pH 8.0], 150 mM NaCl, 0.1% Tween-20) containing 5% skim milk and incubated with primary antibodies (antibodies listed in Supplementary Table 4) diluted in the same buffer overnight at 4 °C. The next day, membranes were washed five times with 1× TBS-T and incubated with secondary antibodies for 1 h at 25 °C, followed by five washes. Proteins were detected using an ECL kit (Thermo Fisher Scientific) and visualized on X-ray film (AGFA, Mortsel, Belgium).

For the co-IP assay, cell lysates were pre-cleared with 20 μL Dynabeads (#10004D, #10002D; Invitrogen) for 4 h and incubated overnight with 20 μL Dynabeads and 5–10 μg antibodies or IgG at 4 °C in a rotary mixer. Beads were extensively washed with cell lysis buffer, and co-IP materials were eluted with loading dye and analyzed by SDS-PAGE.

### In vivo xenograft experiments

Xenograft tumors were generated in 4-week-old female BALB/c nude mice (Orient Bio, Seongnam-si, Republic of Korea) by subcutaneously injecting 5.0 × 10^6^ HCT116 cells suspended in 200 μL of 50% Matrigel (#356234; BD Bioscience) (n = 13–15 mice per group). Mice in the DOX-treated tumor group were administered 1 mg/mL of DOX (#D9891; Sigma-Aldrich) and 5% sucrose in drinking water for 2 weeks. Tumor volumes were calculated as follows: tumor volume (mm^3^) = 1/2 (length × width^2^).

Sample size was not predetermined using statistical methods. The number of animals used was based on prior experience. No animals or samples were excluded from the analysis, and no pre-established inclusion or exclusion criteria were applied. Animals were assigned to experimental groups based on genotype, and no method of randomization was used. Investigators were not blinded to group allocation during the experiments or outcome assessment.

### ChIP-seq and ChIP-qPCR

Cells (2 × 10⁷) were crosslinked with 1% formaldehyde for 20 min at 25 °C, and the reaction was stopped by adding 0.125 M glycine for 5 min. Cells were lysed with 1 mL of cell lysis buffer (10 mM Tris-Cl [pH 8.0], 10 mM NaCl, 0.2% NP-40, protease inhibitors) and incubated for 30 min at 4°C in a rotary mixer. Nuclei were isolated by centrifugation at 853 × *g* for 5 min. Pellets were resuspended in 500 μL of nucleus lysis buffer (50 mM Tris-Cl [pH 8.0], 10 mM EDTA, 1% SDS, protease inhibitors) and incubated for 15 min at 4 °C. Chromatins were digested with MNase (#LS004797; Worthington Biological Corporation, Lakewood, NJ, USA) for 20 min and sheared to 200–400 bp fragments using a QSonica Q500 sonicator. Sonicated chromatin was diluted with 2.5 mL IP dilution buffer (20 mM Tris-Cl [pH 8.0], 150 mM NaCl, 2 mM EDTA, 0.01% SDS, 1% Triton X-100) and incubated with 5–10 μg of antibodies (antibodies listed in Supplementary Table 4) and 50 μL Protein A or G Dynabeads overnight at 4 °C in a rotary mixer. Immunoprecipitated beads were washed twice with low-salt wash buffer (20 mM Tris-Cl [pH 8.0], 150 mM NaCl, 2 mM EDTA, 0.1% SDS, 1% Triton X-100), once with high-salt wash buffer (20 mM Tris-Cl [pH 8.0], 500 mM NaCl, 2 mM EDTA, 0.1% SDS, 1% Triton X-100), once with LiCl wash buffer (10 mM Tris-Cl [pH 8.0], 0.25 M LiCl, 1 mM EDTA, 1% NP-40, 1% Na-deoxycholate), and twice with 1× TE (10 mM Tris-Cl [pH 8.0], 1 mM EDTA). Chromatin was eluted with 300 μL elution buffer (100 mM sodium bicarbonate, 1% SDS). RNase A (Sigma-Aldrich) and 0.25 M NaCl were added, and crosslinks were reversed by incubation overnight at 65 °C. The next day, 100 μg Proteinase K (Invitrogen) was added and incubated at 65 °C for 4 h. Samples were extracted with phenol–chloroform–isoamyl alcohol (#P2069; Sigma-Aldrich) and eluted in 50 μL of 1× TE. ChIP and input DNA libraries were prepared using the NEBNext Ultra II DNA Library Prep Kit (#E7645; New England Biolabs) according to the manufacturer’s instructions.

For ChIP-qPCR analysis, values from immunoprecipitated samples were normalized to input DNA. Primer sequences used for ChIP-qPCR are listed in Supplementary Table 3.

### ATAC-seq

ATAC-seq was performed as previously described [54, 55]. Fifty thousand cells were lysed with 50 μL RSB buffer (10 mM Tris-Cl [pH 7.4], 10 mM NaCl, 3 mM MgCl₂) containing 0.1% NP-40, 0.1% Tween-20, and 0.01% digitonin and incubated for 5 min at 4 °C. After lysis, 1 mL cold RSB buffer with 0.1% Tween-20 was added and mixed by inverting the tubes three times. After centrifugation at 379 × *g* for 10 min, nuclei were resuspended in 50 μL Illumina 1× TD buffer with 2.5 μL Tn5 Transposase and incubated for 30 min at 37 °C in a thermomixer at 68 × *g*. DNA was purified using a QIAGEN MinElute PCR purification kit (Hilden, Germany) and amplified with Nextera sequencing primers and NEBNext high-fidelity 2× PCR master mix (#M0541S; New England Biolabs) for 15 cycles. Purified PCR products were deep sequenced (paired-end 2 × 75 bp) on a NextSeq500 platform (Illumina).

### Data processing for ATAC-seq

Paired-end libraries were generated for ATAC-seq. Reads were aligned to the hg19 reference genome using Bowtie after adapter trimming with Trimmomatic. Mapped reads were filtered to remove mitochondrial and duplicate reads with Samtools and used for downstream analyses. Peaks were called using the findPeaks function in Homer with the parameter (-style factor). After merging peaks across samples, differential regions were identified using getDiffExpression.pl with a cutoff of |FC | > 2 and p < 0.01. Gene annotation was performed using annotate.pl. For motif analysis, findMotifsGenome.pl was used with the (-size) parameter to identify candidate TFs.

### Data processing for ChIP-seq

Single-end libraries were generated for SMARCB1, INO80, KLF4, and RNA Pol II-S2P ChIP-seq. Reads were aligned to the hg19 reference genome using Bowtie after adapter trimming with Trimmomatic. Mapped reads were filtered to remove mitochondrial and duplicate reads with Samtools and used for downstream analyses. Peaks were called using Homer’s findPeaks function with the input file as control and the parameter (-style factor).

### Data processing for RNA-seq

Paired-end libraries were generated for RNA-seq. Reads were aligned to the hg19 reference genome using STAR after adapter trimming with Trimmomatic. The mapped files were used as input for Homer’s MaketagDirectory.pl for downstream analyses. DEGs were identified from raw counts using getDiffExpression.pl with a cutoff of false discovery rate < 0.05 and |FC | > 1.5. Transcripts per million values were used for heatmap visualization with Pheatmap in R. Hallmark pathways from MsigDB were downloaded for functional enrichment analysis.

### Statistical analysis

No statistical methods were used to predetermine sample size. Statistical tests used for each figure are indicated in the corresponding figure legends. Data distribution was assessed using the Shapiro–Wilk test to confirm normality, and variance equality between groups was evaluated using the F-test. Statistical significance was determined using an unpaired two-tailed Student’s *t*-test. Error bars represent the mean ± standard deviation (SD). A *p*-value < 0.05 was considered statistically significant. All analyses were performed using GraphPad Prism version 8 (GraphPad Software, Boston, MA, USA).

### Illustration tools

The graphical abstract was created with BioRender (https://www.biorender.com/).

## Supplementary information

Supplementary Material

Original western blots

## Data Availability

All the sequencing data generated in this study can be accessed through the European Nucleotide Archive (ENA) using the project accession code PRJEB82643.
