# Supplementary material for: ACTL6A depletion induces KLF4-mediated anti-tumorigenic effects in colorectal cancer
Source: Cell Death Dis. 2025 Aug 28;16(1):653. doi: 10.1038/s41419-025-07946-w (PMC12394641; doi:10.1038/s41419-025-07946-w)
Supplement: Supplementary file 1 — Supplementary Material [file 41419_2025_7946_MOESM1_ESM.docx]

**Supplementary Figures and Figure legends**

**
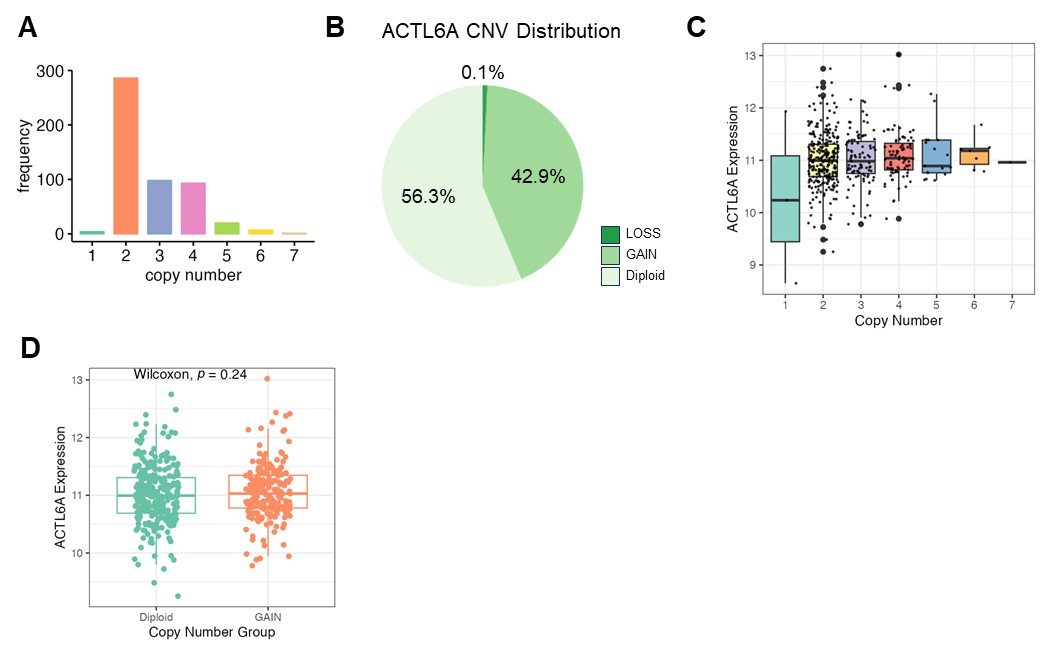
**

**Supplementary Figure 1. Copy number profile and expression status of *ACTL6A* in CRC tissues**

1. Bar plot indicating the distribution of *ACTL6A* copy number groups based on The Cancer Genome Atlas (TCGA)-colon adenocarcinoma (COAD) gene copy numbers.
2. Pie chart illustrating the percentage of each copy number group. Samples with >2 copy numbers of *ACTL6A* were categorized as GAIN. Samples with <2 copy numbers of *ACTL6A* were categorized as LOSS.
3. Box plot of expression among the tumor samples grouped according to copy number. The Kruskal–Wallis test was used to compare group differences. Expression values were determined using variance stabilizing transformation and the DESeq2 package.
4. Box plot of expression between normal and tumor samples grouped according to copy number. The Kruskal–Wallis test was used to compare group differences. Expression values were determined using VST and the DESeq2 package.

**
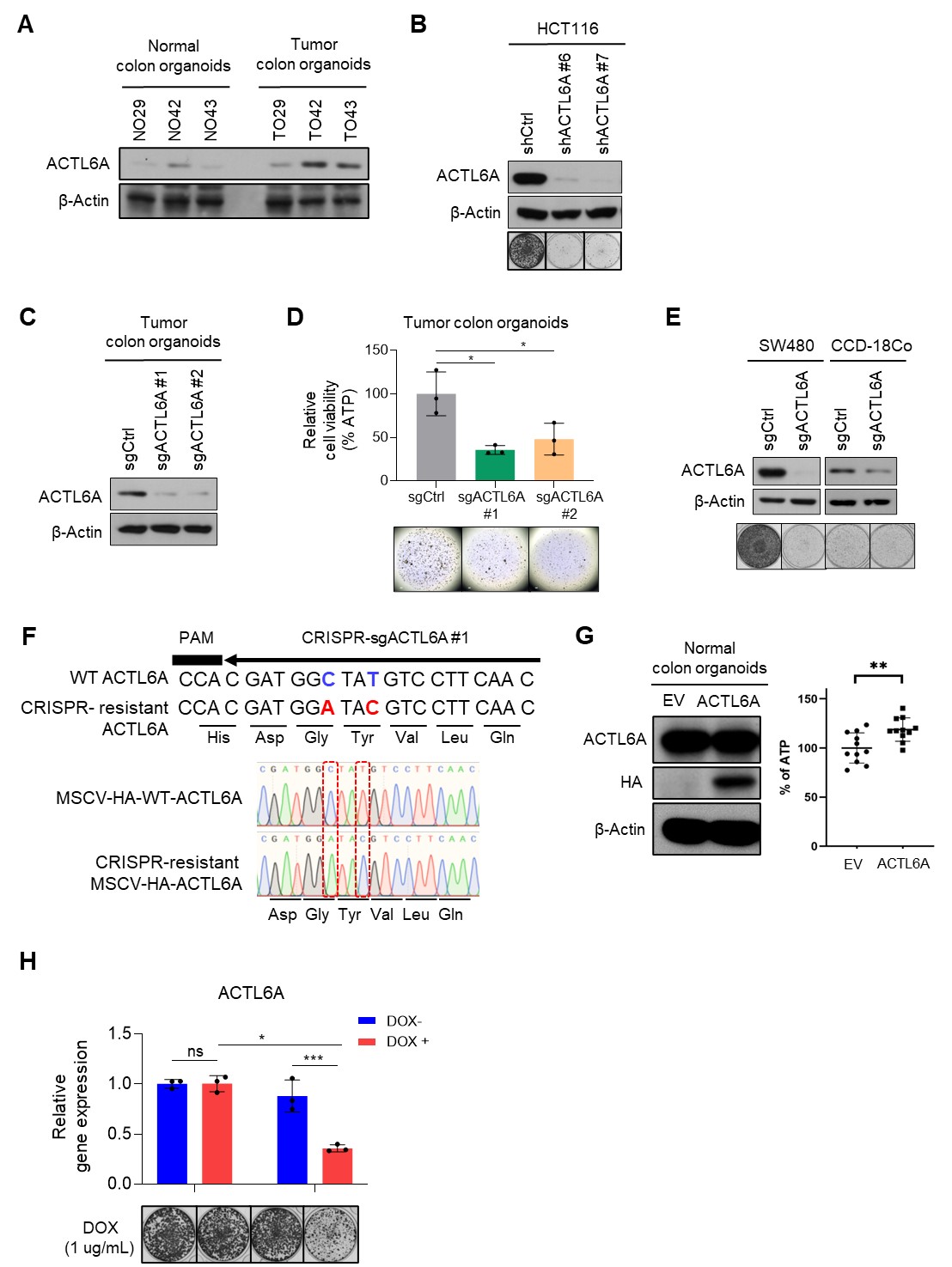
**

**Supplementary Figure 2. ACTL6A depletion and overexpression in CRC cells and organoids derived from patients with CRC (PDOs)**

1. Protein expression levels of ACTL6A in three pairs of CRC–PDOs (TO29, TO42, and TO43) and matched normal colon organoids (NO29, NO42, and NO43).
2. Western blotting of ACTL6A in HCT116 cells transduced with the indicated shRNAs, shCtrl, or two individual shRNAs against ACTL6A. β-Actin was used as the loading control.
3. Western blotting of ACTL6A in CRC PDOs transduced with sgCtrl or sgACTL6As. β-Actin was used as the loading control.
4. Relative viability of PDO cells after ACTL6A depletion was determined using a CellTiter-Glo 3D Cell Viability Kit (top). ATP levels (y-axis) were measured. Representative images of PDO formation and growth inhibition (bottom). n = 3 biological replicates. Scale bar: 200μm. **p <* 0.05.
5. Western blotting of ACTL6A in SW480 and CCD-18Co cells transduced with sgCtrl or sgACTL6A (top). β-Actin was used as the loading control. Colony formation assay of SW480 and CCD-18Co cells transduced with sgCtrl or sgACTL6A (bottom).
6. Design of ACTL6A knockout-resistant vectors. Sanger sequencing of the WT and CRISPR-resistant ACTL6A constructs.
7. Western blotting of the indicated antibodies in normal colon PDOs transduced with empty vector (EV) or HA-tagged ACTL6A expressing vector (left) and cell viability assay (right). ***p <* 0.01.
8. Normalized mRNA expression of *ACTL6A* in HCT116 cells with doxycycline (DOX)-inducible shRNA targeting Ctrl (GFP) or ACTL6A. mRNA levels were analyzed using quantitative reverse transcription-polymerase chain reaction (RT-qPCR) (top) and colony formation assays (bottom). n = 3 biological replicates. **p <* 0.05, ****p <* 0.001, ns, not significant.

**
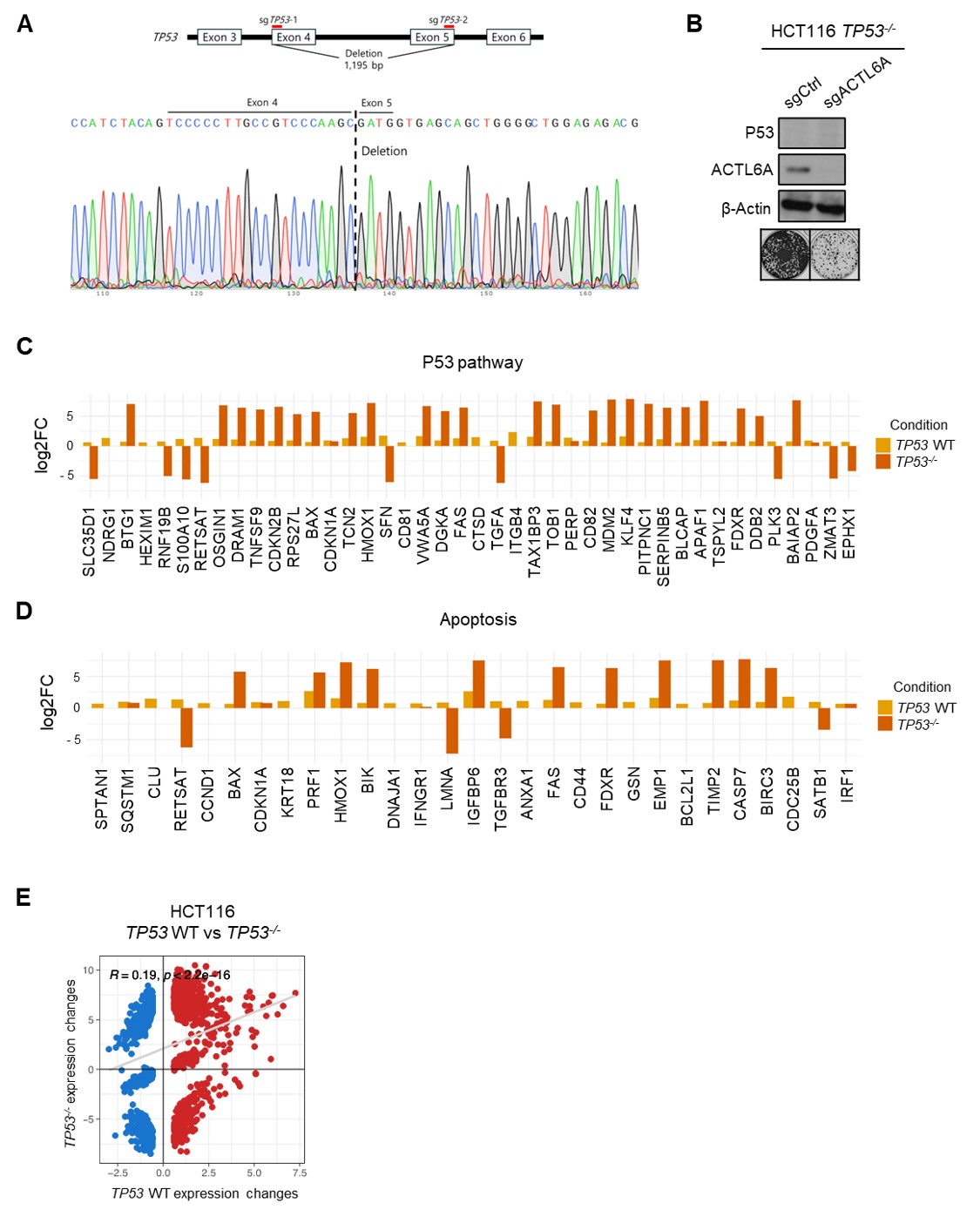
**

**Supplementary Figure 3. ACTL6A depletion induces cell death independent of P53**

1. Sanger sequencing of HCT116 *TP53^-/-^* cells showing a 1,195 bp deletion in *TP53.*
2. Western blotting of P53 and ACTL6A in HCT116 *TP53^-/-^* cells transduced with sgCtrl or sgACLT6A. β-Actin was used as the loading control (top). Colony formation assay of the HCT116 *TP53^-/-^* cells transduced with sgCtrl or sgACLT6A (bottom).
3. Bar plot illustrating changes in the expression (log2FC) of P53-related upregulated genes in HCT116 *TP53* WT and *TP53^-/-^* cells upon ACTL6A depletion.
4. Bar plot illustrating changes in the expression (log2FC) of apoptosis-related genes in HCT116 *TP53* WT and *TP53^-/-^* cells upon ACTL6A depletion.
5. Correlation plot illustrating the changes in expression (log2FC) in HCT116 *TP53* WT and *TP53^-/-^* cells. Red and blue indicate the upregulated and downregulated genes, respectively. Pearson’s method was used to calculate the correlation coefficients.

**
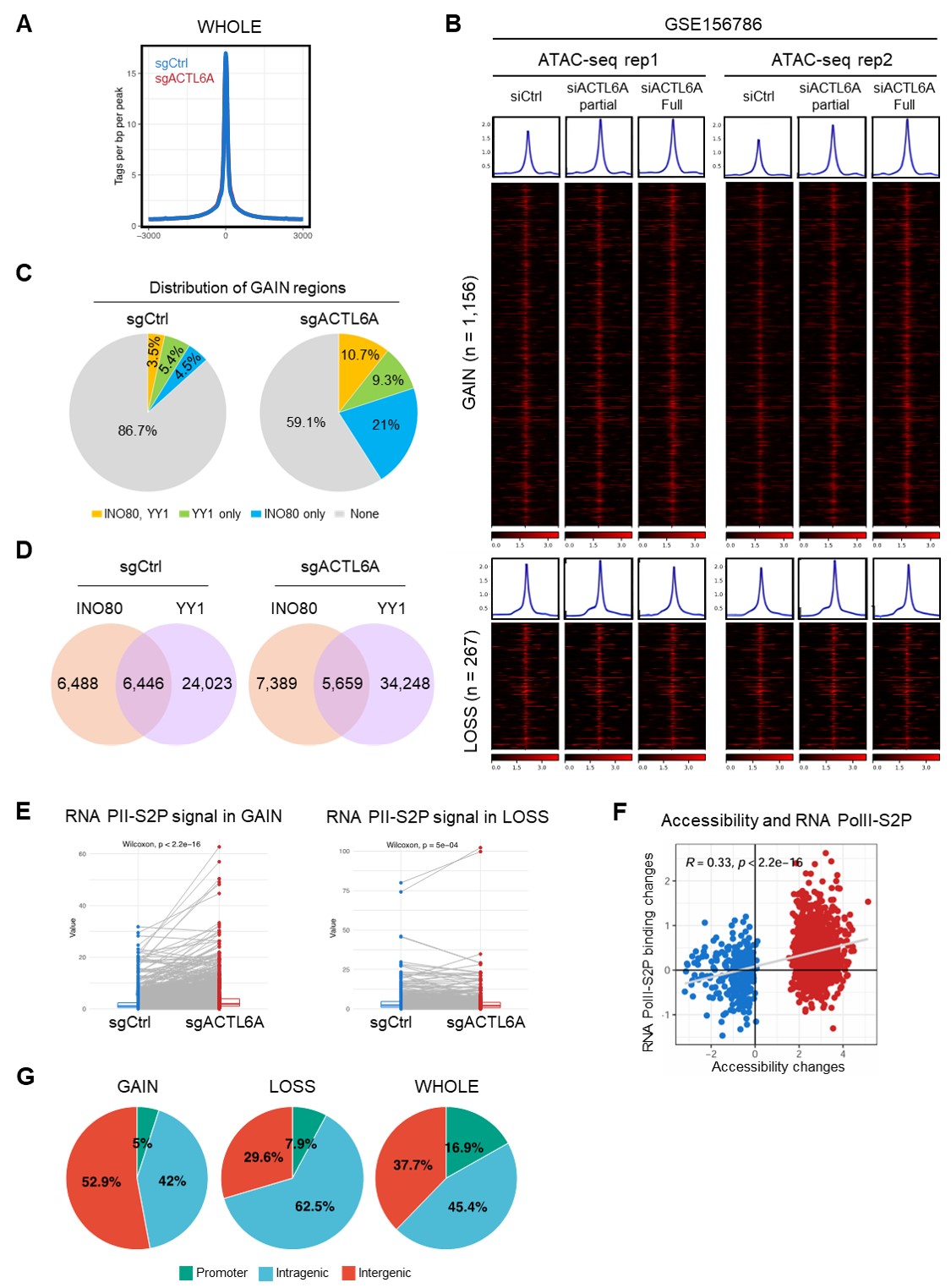
**

**Supplementary Figure 4. Evaluation of chromatin accessibility, binding patterns of INO80 and YY1, and RNA PII binding after ACTL6A depletion**

1. Histogram demonstrating tags per bp per peak from −3,000 bp upstream to +3,000 bp downstream of the identified WHOLE after ACTL6A depletion in HCT116 cells compared with the WT, based on ATAC-seq (n = 3).
2. Heatmaps demonstrating the GSE156786 ATAC-seq reads from −3,000 bp upstream to +3,000 bp downstream of the identified GAIN and LOSS regions after partial and complete siBAF53 treatment in FaDu cells, a pharyngeal squamous cell carcinoma cell line.
3. Pie charts summarizing the binding patterns of YY1 and INO80 within GAIN regions under sgCtrl and sgACTL6A conditions.
4. Venn diagram showing the overlap of YY1 and INO80 binding peaks genome-wide under sgCtrl and sgACTL6A conditions.
5. RNA PII-S2P ChIP-seq signal changes following ACTL6A depletion. A significant increase in RNA PII-S2P binding was observed at GAIN regions (Wilcoxon paired test, *p* < 2.2e-16) (top), while a significant decrease was detected at LOSS regions (bottom) upon ACTL6A depletion (Wilcoxon paired test, *p* = 5e-04).
6. Correlation plot illustrating alterations in accessibility and RNA PII-S2P enrichment. Red and blue indicate the GAIN and LOSS regions, respectively. Pearson’s analysis was used to calculate correlation coefficients.
7. Pie charts indicating the genomic coordinates of the identified GAIN, LOSS, and WHOLE regions.

**
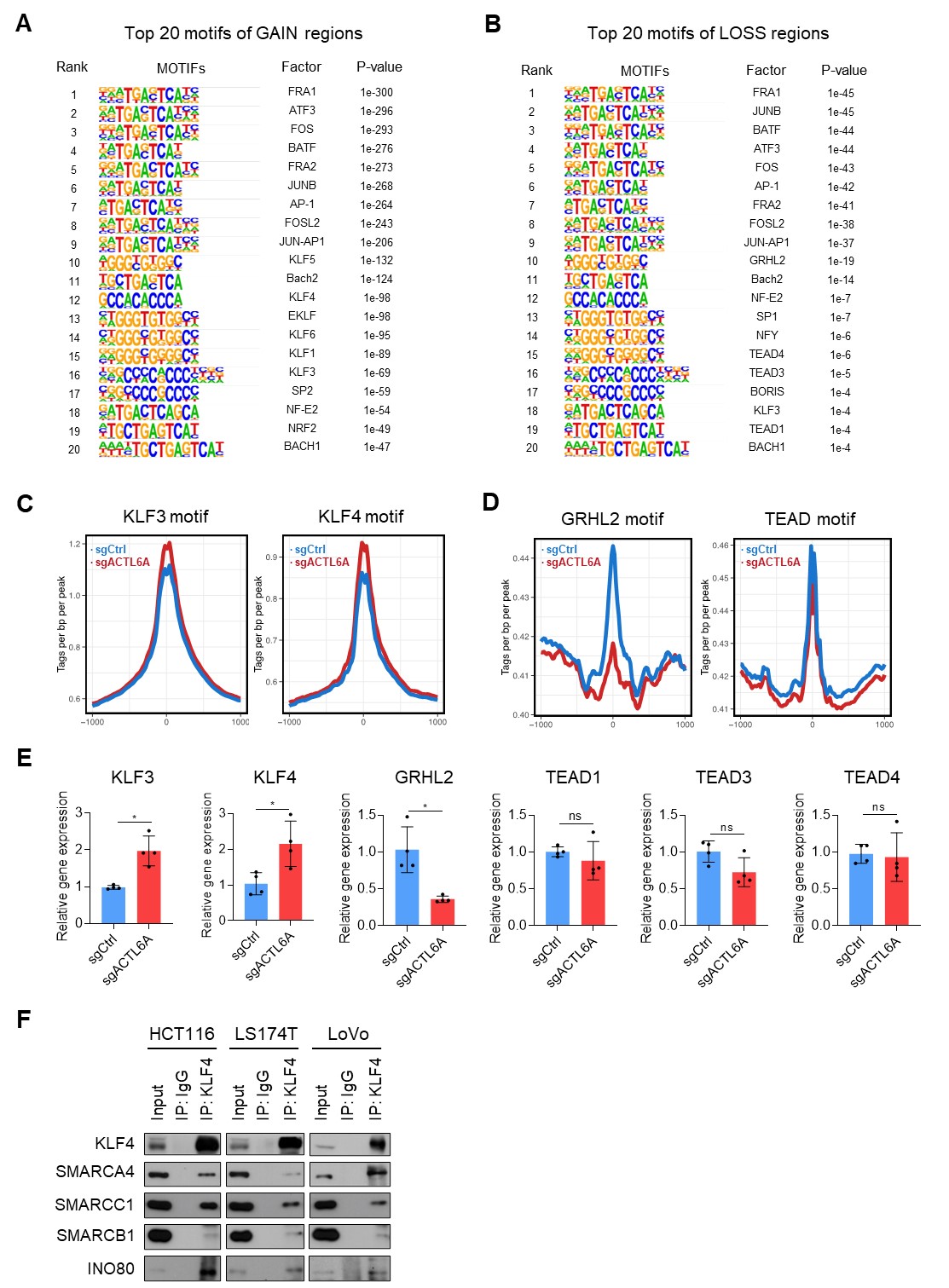
**

**Supplementary Figure 5. Motif analysis of GAIN and LOSS regions and interaction between KLF4 and SWI/SNF, INO80 complex subunits**

1. Top 20 ranked motif analysis results for the GAIN (left) and LOSS (right) regions.
2. Average plots indicating tags per bp per peak from −1,000 bp upstream to +1,000 bp downstream of the KLF3 and KLF4 motifs in the genome.
3. Average plots indicating tags per bp per peak from −1,000 bp upstream to +1,000 bp downstream of the GRHL2 and TEAD motifs in the genome.
4. Normalized mRNA expression of the indicated genes in HCT116 cells transduced with sgCtrl or sgACTL6A was measured using RT-qPCR. n = 4 biological replicates. **p <* 0.05, ns, not significant.
5. Co-IP experiments using the KLF4 antibody on whole-cell extracts from HCT116, LS174T, and LoVo cells. Western blotting results of ACTL6A (SWI/SNF and INO80 complex subunit), SMARCA4, SMARCC1, SMARCB1 (SWI/SNF complex subunits), INO80 (INO80 complex subunit).

**
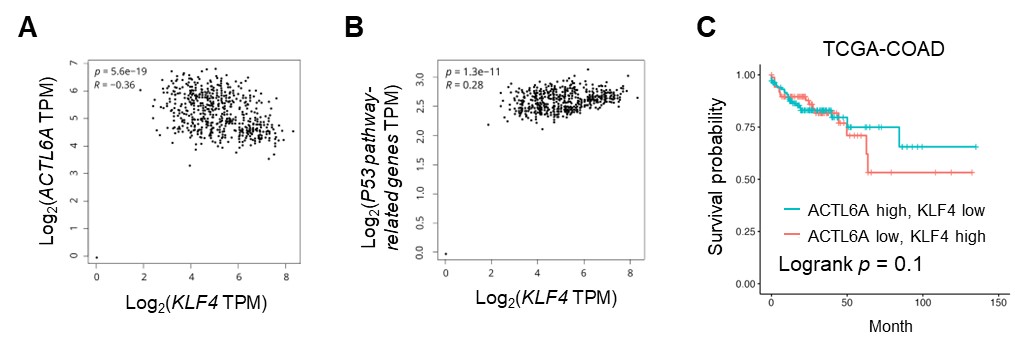
**

**Supplementary Figure 6. Correlation of ACTL6A and P53 pathway related genes with KLF4, survival analysis of KLF4 and ACTL6A**

1. Correlation between *KLF4* and *ACTL6A* mRNA expression in CRC tissues was analyzed using the TCGA and GTEx datasets available in GEPIA 2. R represents Pearson’s correlation coefficient.
2. Correlation between *KLF4* and mRNA expression levels of P53 pathway-related genes (*SERPINB5, DGKA, S100A10, TOB1, BAIAP2, CDKN2B, CTSD)* in CRC tissues was analyzed using the TCGA and GTEx datasets available in GEPIA 2. R is Pearson’s correlation coefficient.
3. Survival analysis of *ACTL6A* high/*KLF4* low and *ACTL6A* low/*KLF4* high COAD in samples from patients with colon adenocarcinoma from TCGA. *p* = 0.1.

|  | |  | **sgRNAs and shRNAs sequences** |
| --- | --- | --- | --- |
| Target |  | Sequence (5' → 3') | |
| sgACTL6A-1 | F | CACCGGTTGAAGGACATAGCCATCG | |
|  | R | AAACCGATGGCTATGTCCTTCAACC | |
| sgACTL6A-2 | F | CACCGTGCCAAGACCTCGTAACCTG | |
|  | R | AAACCAGGTTACGAGGTCTTGGCAC | |
| sgKLF4-1 | F | CACCGGTGGTGGCGCCCTACAACGG | |
|  | R | AAACCCGTTGTAGGGCGCCACCACC | |
| sgKLF4-2 | F | CACCGGAAGGATCTCGGCCAATTTG | |
|  | R | AAACCAAATTGGCCGAGATCCTTCC | |
| sgTP53-1 | F | CACCGCCCCTTGCCGTCCCAAGCAA | |
|  | R | AAACTTGCTTGGGACGGCAAGGGGC | |
| sgTP53-2 | F | CACCGGAGCGCTGCTCAGATAGCGA | |
|  | R | AAACTCGCTATCTGAGCAGCGCTCC | |
| CRISPR-resistant ACTL6A #1 | F | caatgccttgttgaaggacgtatccatcgtggactggaattg | |
|  | R | caattccagtccacgatggatacgtccttcaacaaggcattg | |
| shACTL6A-6 |  | CCGGGCTTTCCTTGAAATGCACTTACTCGAGTAAGTGCATTTCAAGGAAAGCTTTTT | |
| shACTL6A-7 |  | CCGGCGGTACTTCAAGTGTCAGATTCTCGAGAATCTGACACTTGAAGTACCGTTTTT | |
| sgKLF4-GAIN-1 | F | CACCGTACCAGGAGTGTTTTCAAAG | |
|  | R | AAACCTTTGAAAACACTCCTGGTAC | |
| sgKLF4-GAIN-2 | F | CACCGAAGGATAGAAGGGCCTAGGA | |
|  | R | AAACTCCTAGGCCCTTCTATCCTTC | |

**Supplementary Table 1 – sgRNAs and shRNAs sequences**

**Supplementary Table 2 – RT-qPCR primers**

| **RT-qPCR oligonucleotide sequences** | | |
| --- | --- | --- |
| Gene | Sequence (5' → 3') | |
| ACTL6A | F | CAGAGGCACCGTGGAATACT |
|  | R | AGGACATAGCCATCGTGGAC |
| KLF4 | F | ACCCTGGGTCTTGAGGAAGT |
|  | R | ACGATCGTCTTCCCCTCTTT |
| SERPINB5 | F | GGTGGGGATTCCATAGAGGT |
|  | R | AATCGGCATCCACAGAAAAG |
| DGKA | F | CTCCAACCTCTGGGGTGATA |
|  | R | TTTTGTGGTGTGGAAGGTGA |
| S100A10 | F | GGGCTTCCAGAGCTTCTTTT |
|  | R | CTTCTATGGGGGAAGCTGTG |
| TOB1 | F | ACAGCCCCCTTAACCTCAGT |
|  | R | GCCCGTGCATTTTAACTTGT |
| BAIAP2 | F | CAGTAGGGCCAGAACACCAT |
|  | R | TTGAGGCTTAGAACGCTGGT |
| CDKN2B | F | GACCGGGAATAACCTTCCAT |
|  | R | CACCAGGTCCAGTCAAGGAT |
| CTSD | F | GACACAGGCACTTCCCTCAT |
|  | R | CTCTGGGGACAGCTTGTAGC |

**Supplementary Table 3 – ChIP-qPCR primers**

| **ChIP-qPCR oligonucleotide sequences** | | |
| --- | --- | --- |
|  | Sequence (5' → 3') | |
| double knockout (DKO) | F | GTGAAACCCCGTCTCTGCTA |
|  | R | CTGGAGTGCAGTGGTGTGAT |
| KLF4-GAIN deletion | F | AGTTCCTGGACAGGAGTGGA |
|  | R | GAGCGGGGAGGTGTATCTTT |

**Supplementary Table 4 – Antibodies used in this study**

| **Antibody** | **Manufacturer** | **Cat. No.** | **Application** |
| --- | --- | --- | --- |
| ACTL6A | Bethyl Laboratories | #A301-391 | WB |
| KLF4 | R&D Systems | #AF3640 | WB |
| KLF4 | Novus Biologicals | #NBP1-83940 | ChIP |
| Cleaved caspase-3 | Cell Signaling Technology | #9661 | WB |
| Cleaved caspase-9 | Cell Signaling Technology | #7237 | WB |
| β-Actin | Santa Cruz Biotechnology | #sc1616 | WB |
| SMARCA4 | Abcam | #ab110641 | WB, ChIP |
| SMARCA4 | Santa Cruz Biotechnology | #sc-17796 | WB |
| SMARCB1 | Bethyl Laboratories | #A301-087A | WB, ChIP |
| SMARCC1 | Santa Cruz Biotechnology | #sc-32763 | WB |
| SS18 | Cell Signaling Technology | #21792 | WB |
| INO80 | Abcam | #ab118787 | WB, ChIP |
| YY1 | Santa Cruz Biotechnology | #sc-7341 | WB |
| YY1 | Active Motif | #61779 | ChIP |
| RNA Polymerase II phospho S2 | Abcam | #ab5095 | ChIP |
| Rabbit IgG | Abcam | #ab37415 | ChIP (Negative control) |
| Mouse IgG | Abcam | #ab37355 | ChIP (Negative control) |
| Goat IgG | Santa Cruz Biotechnology | #sc-2028 | ChIP (Negative control) |
| Rabbit anti-HA | Abcam | #ab9110 | WB |
| Mouse anti-HA | Abcam | #ab18181 | WB |
