# Supplementary material for: ACTL6A depletion induces KLF4-mediated anti-tumorigenic effects in colorectal cancer
Source: Cell Death Dis. 2025 Aug 28;16(1):653. doi: 10.1038/s41419-025-07946-w (PMC12394641; doi:10.1038/s41419-025-07946-w)

Original western blots

Figure 1D

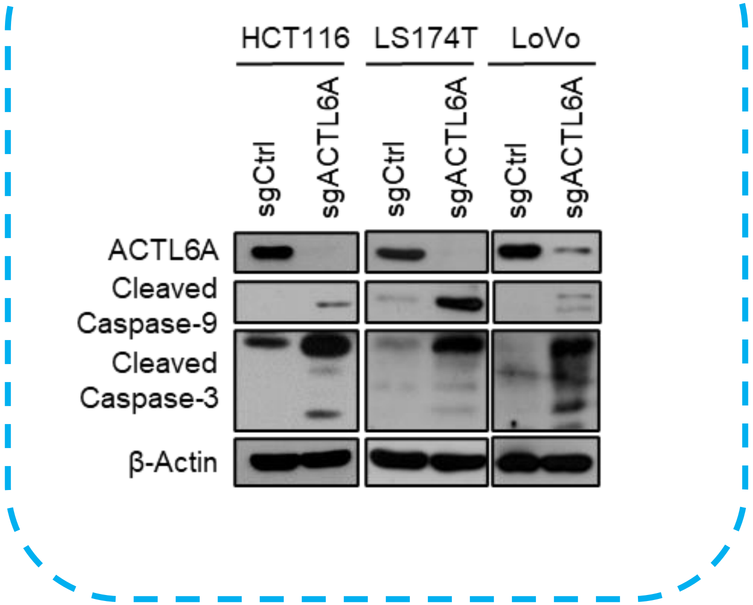

Figure 1D

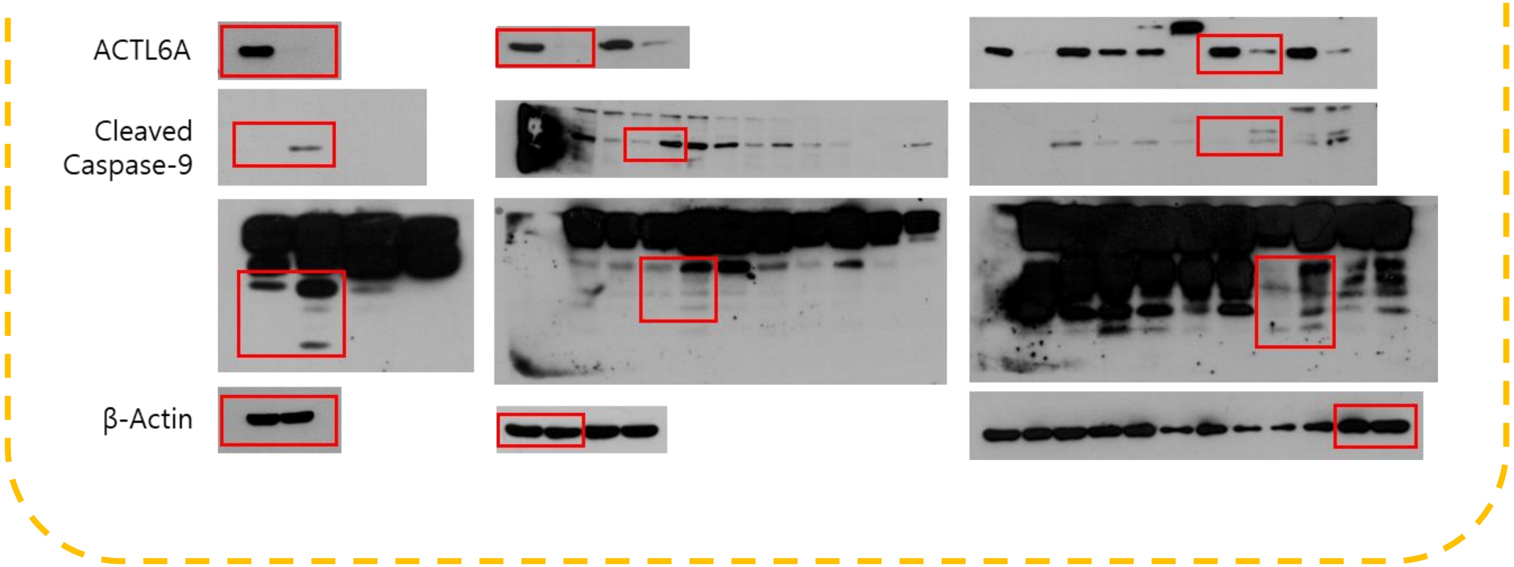

Figure 1F

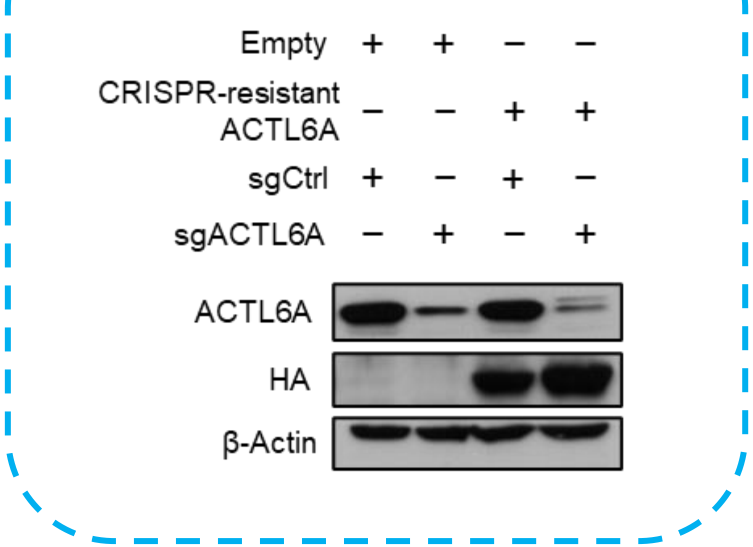

Figure 1F

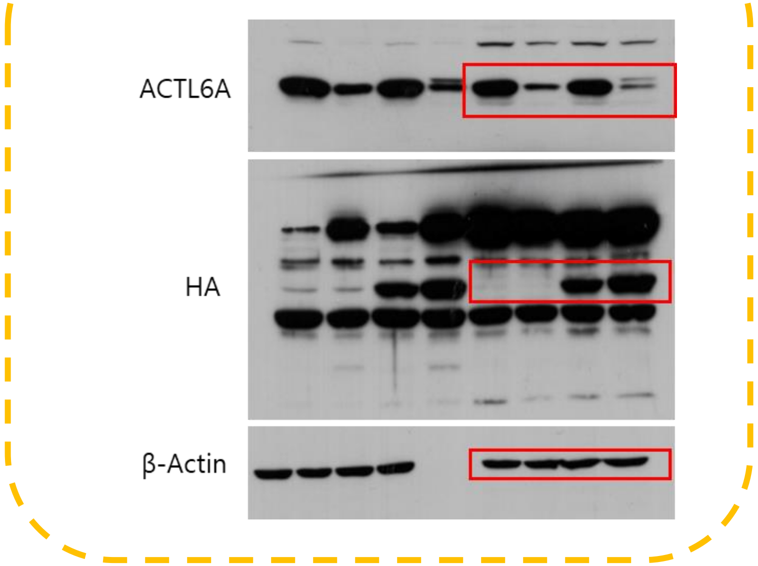

Figure 1G

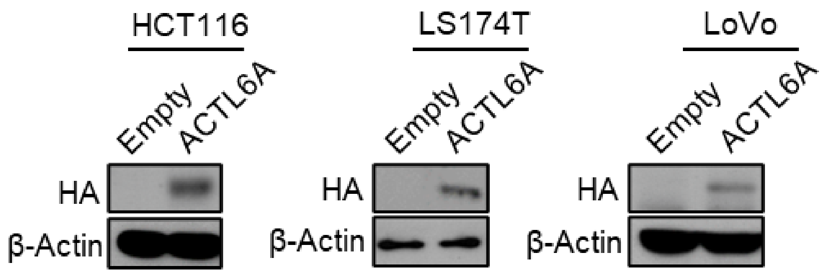

Figure 1G

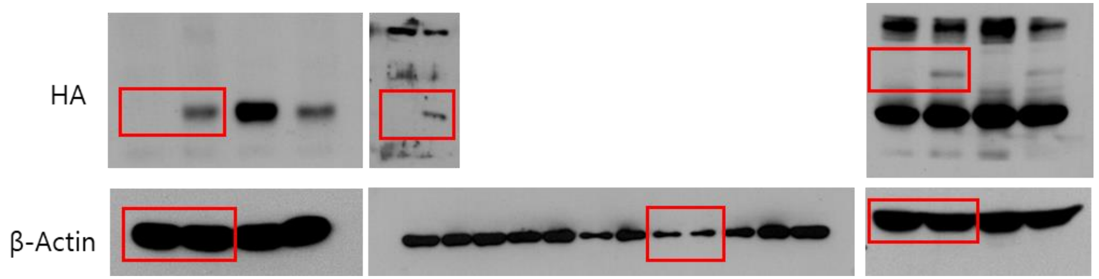

Figure 1I

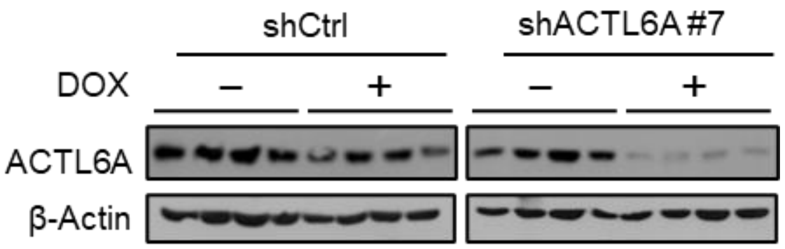

Figure 1I

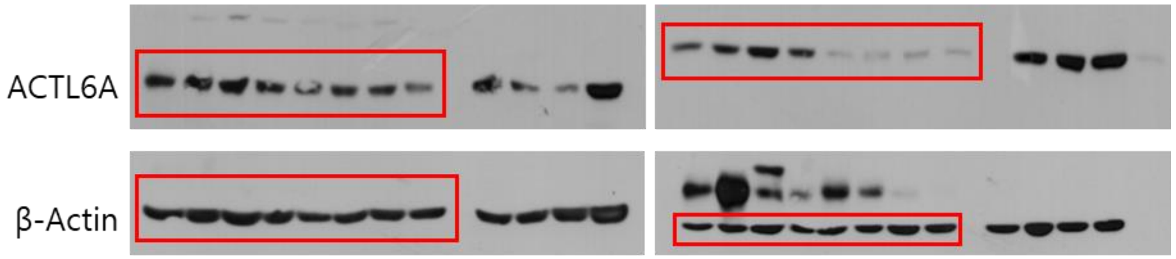

Figure 3A

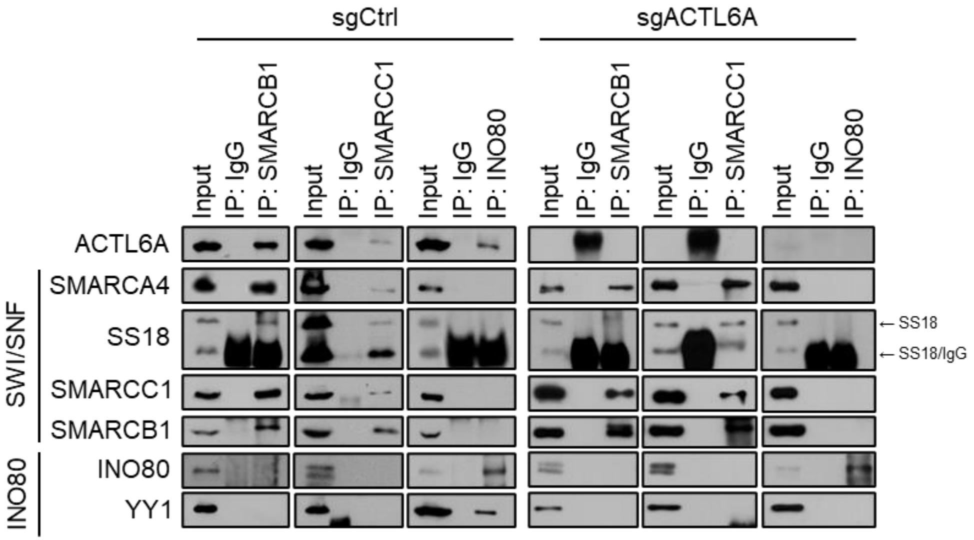

Figure 3A

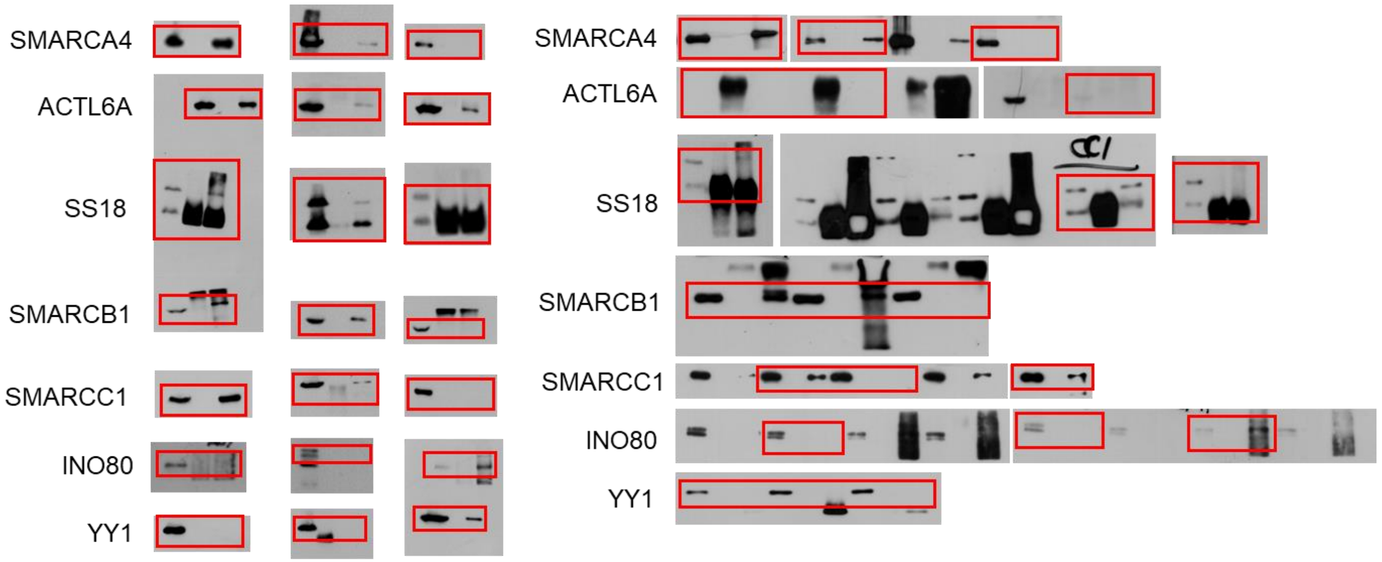

Figure 4H

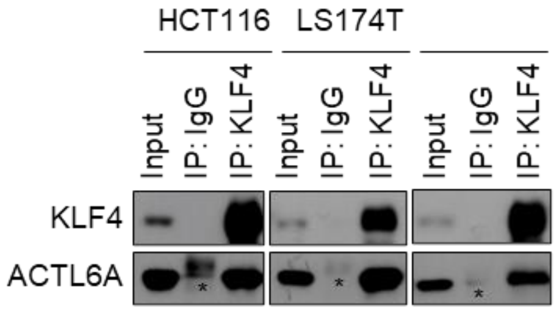

Figure 4H

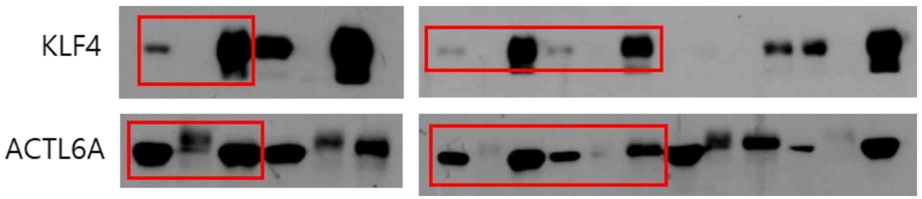

Figure 4I

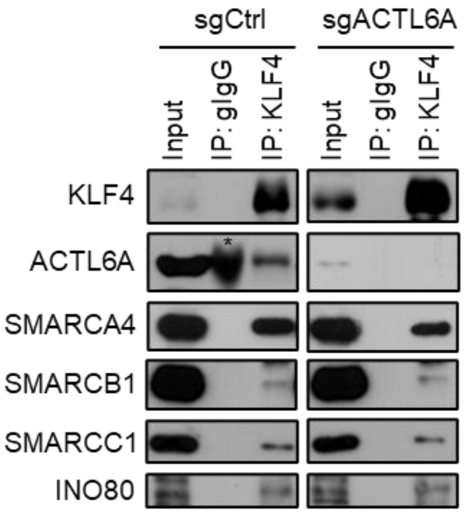

Figure 4I

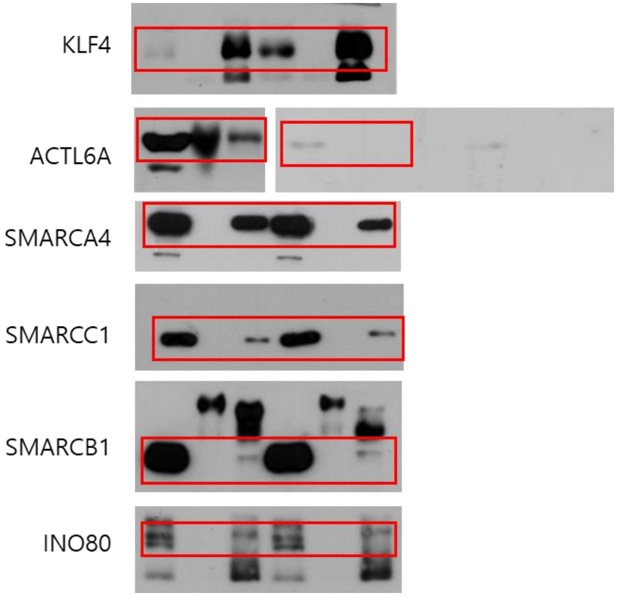

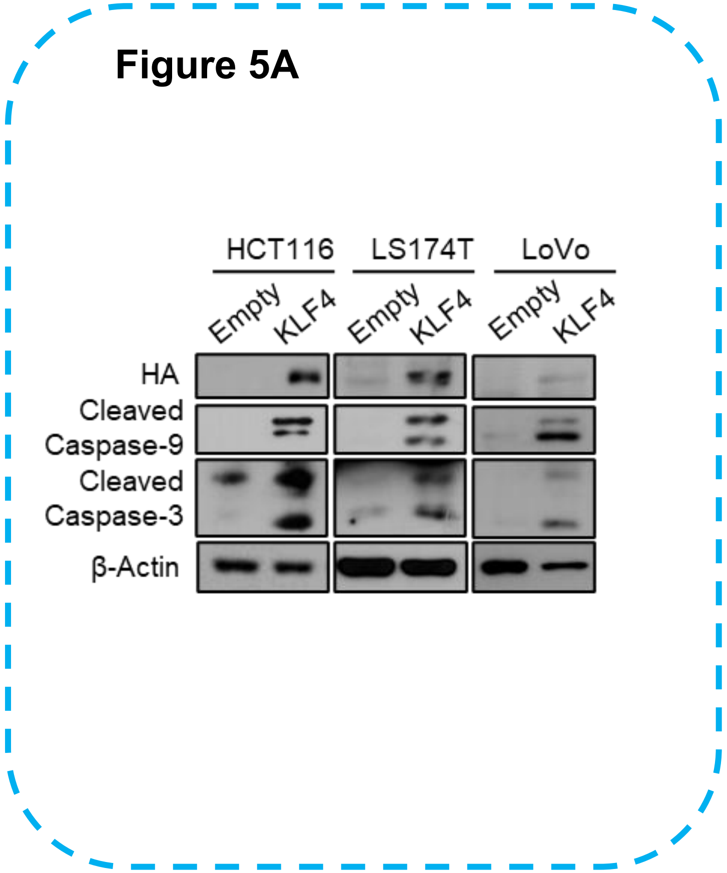
Figure 5A
HCT116
LS174T
LoVo
Empty
KLF4
Empty
KLF4
Empty
KLF4
HA
Cleaved Caspase-9
Cleaved Caspase-3
β-Actin

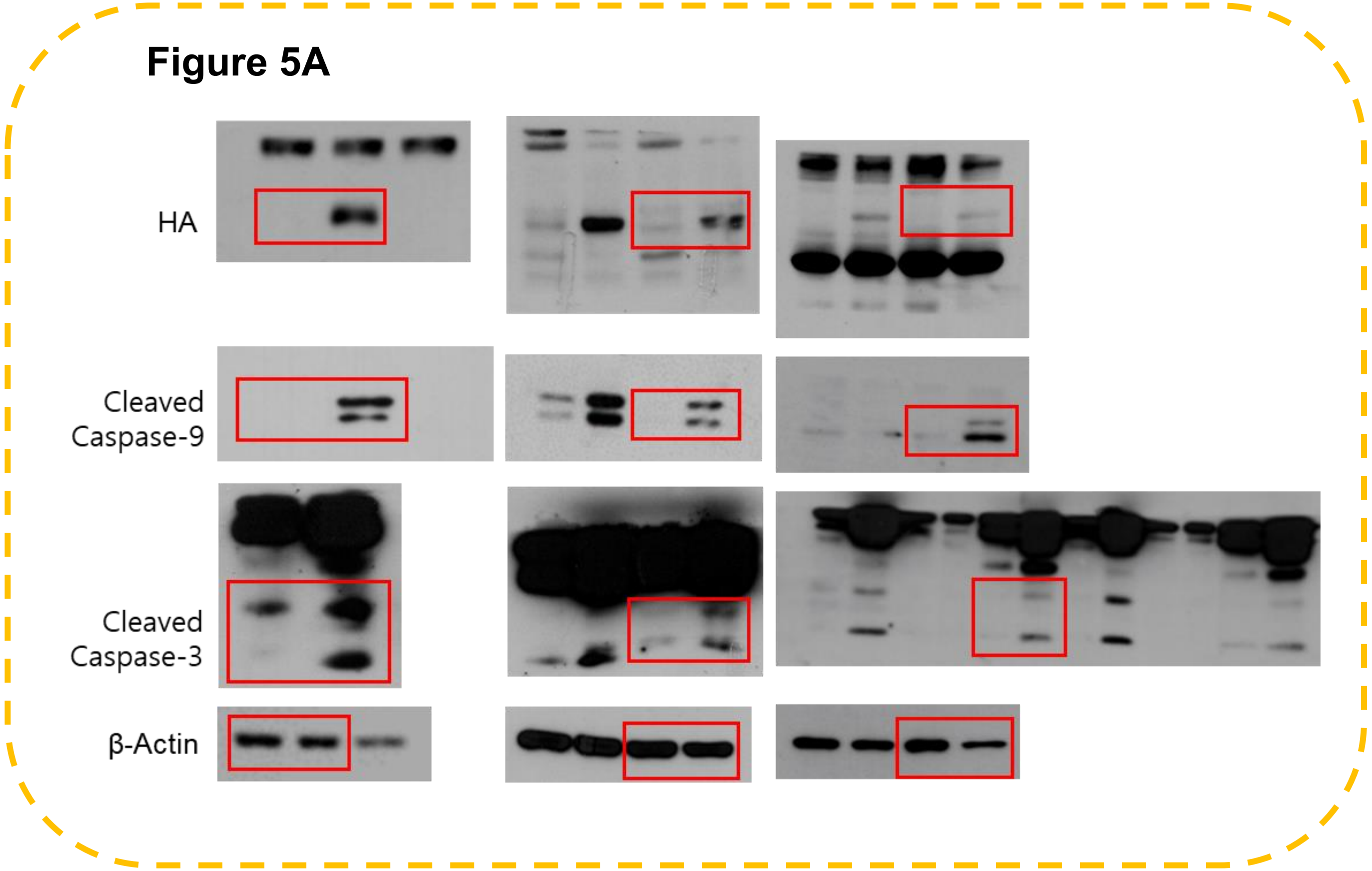
Figure 5A
HA
Cleaved Caspase-9
Cleaved Caspase-3
β-Actin

Figure 5F

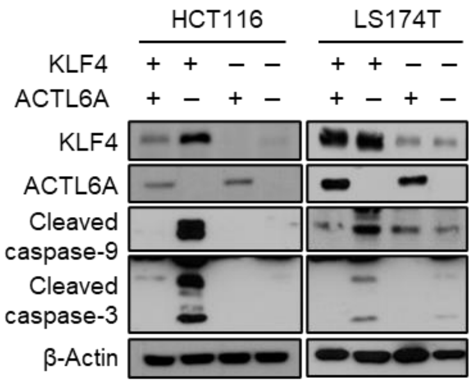

Figure 5F

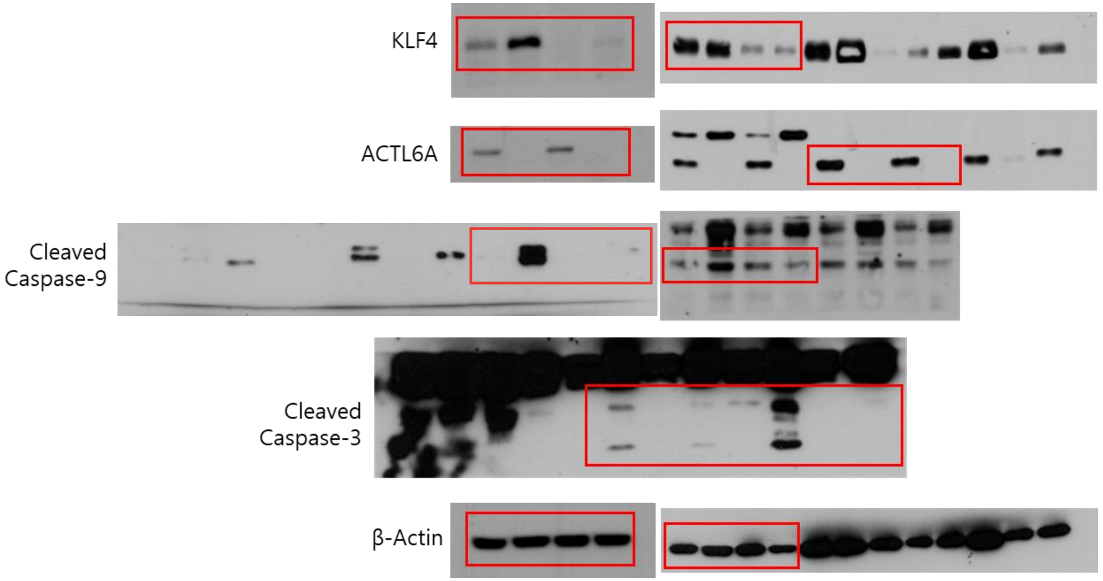

Figure 6H

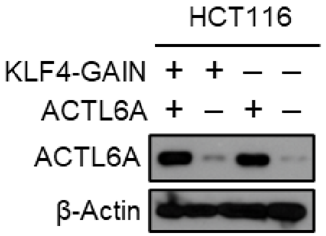

Figure 6H

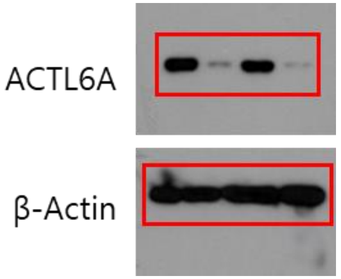

Supple Figure 2A

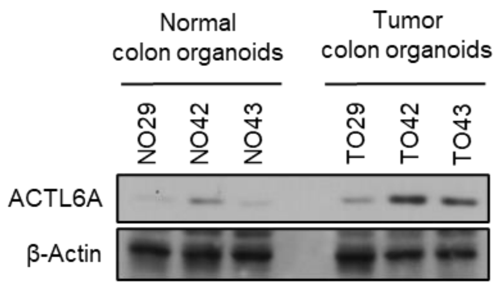

Supple Figure 2B

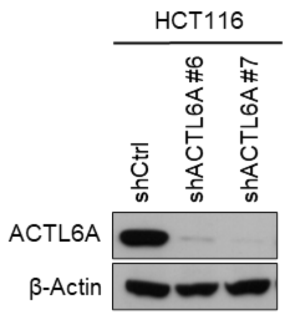

Supple Figure 2C

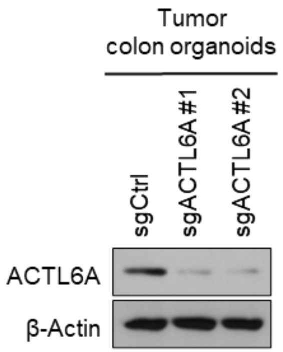

Supple Figure 2A

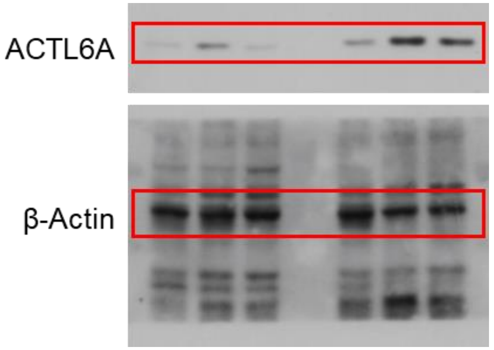

Supple Figure 2B

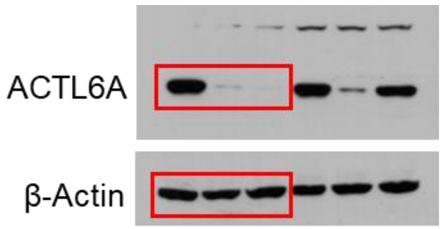

Supple Figure 2C

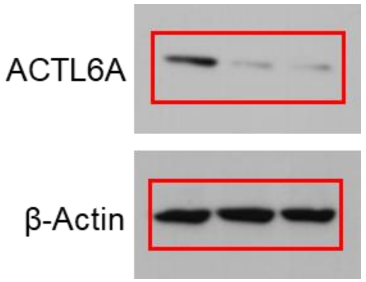

Supple Figure 2E

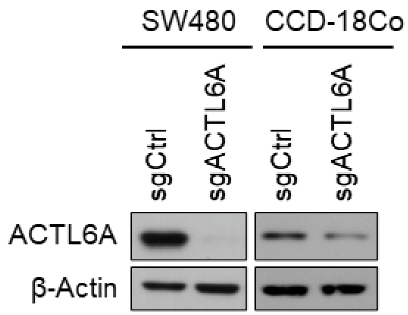

Supple Figure 2E

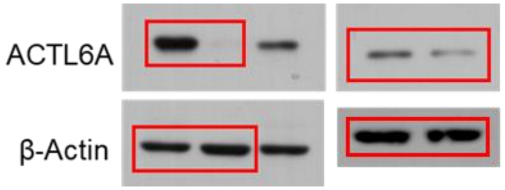

Supple Figure 2G

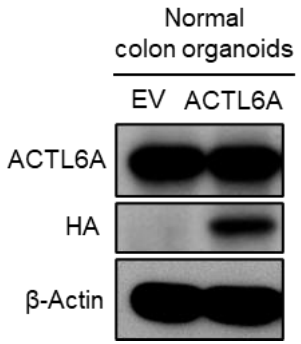

Supple Figure 2G

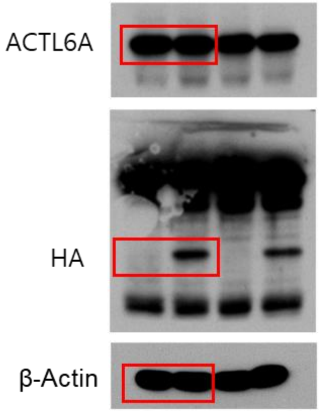

Supple Figure 3B

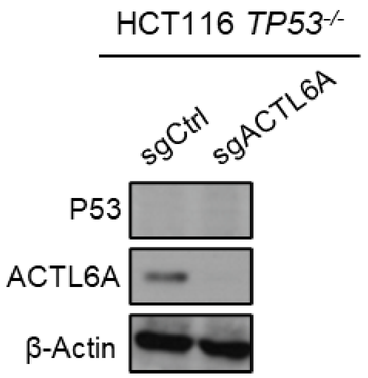

Supple Figure 3B

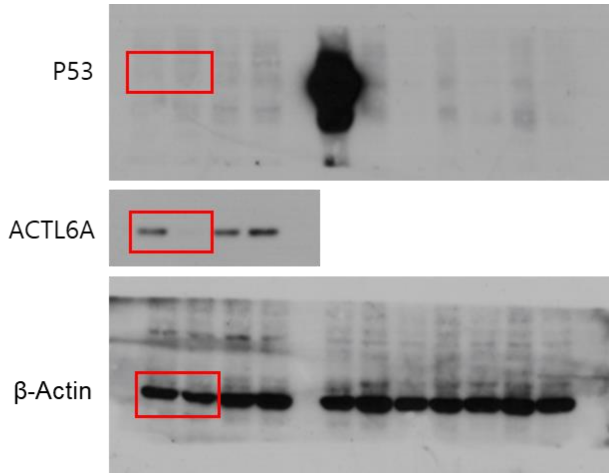

Supple Figure 5F

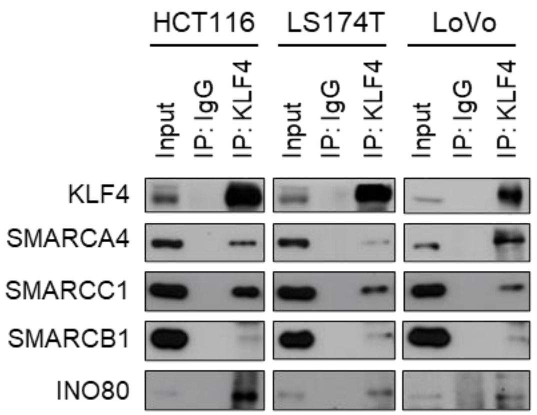

Supple Figure 5F

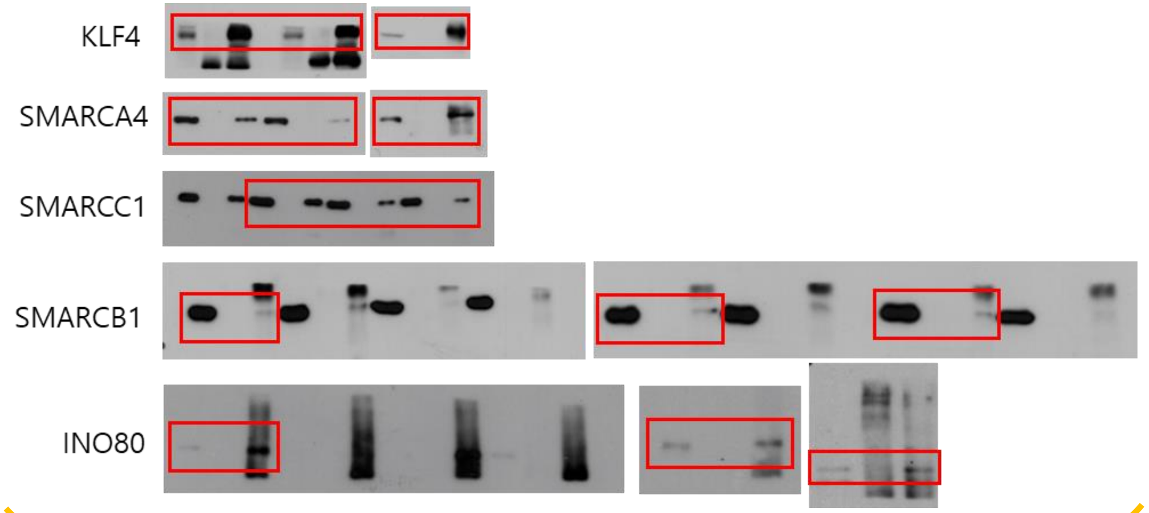

Supplement: Supplementary file 2 — Original western blots [file 41419_2025_7946_MOESM2_ESM.pdf]
